# A novel chimpanzee adenovirus vector vaccine for protection against infectious bronchitis and Newcastle disease in chickens

**DOI:** 10.1186/s13567-025-01528-6

**Published:** 2025-05-16

**Authors:** Chengyao Hou, Ruiqi Ni, Lijun Zhao, Wenjun Yan, Kailu Wang, Qinyuan Chu, Xinggui Chen, Hongning Wang, Xin Yang

**Affiliations:** 1 Key Laboratory of Bio‑Resource and Eco‑Environment of Ministry of Education, College of Life Sciences, Sichuan University, Chengdu, Sichuan China; 2 Animal Disease Prevention and Food Safety Key Laboratory of Sichuan Province, Chengdu, China

**Keywords:** nonreplicating chimpanzee adenovirus, bivalent vaccine, IBV S1 protein, NDV HN protein, poultry immunization

## Abstract

The development of effective poultry vaccines is crucial for maintaining flock health and productivity. In this study, we developed and evaluated a recombinant chimpanzee adenovirus vaccine (PAD-S1-HN) that simultaneously expresses the infectious bronchitis virus (IBV) spike subunit protein S1 and Newcastle disease virus (NDV) hemagglutinin-neuraminidase HN protein. The recombinant virus was successfully rescued in HEK293 cells, and transmission electron microscopy confirmed its typical adenoviral morphology. The expression of the IBV S1 and NDV HN proteins was validated by indirect immunofluorescence assay and western blotting. The vaccine demonstrated genetic stability over multiple passages and exhibited growth kinetics similar to those of the empty chimpanzee adenovirus vector. In animal trials, PAD-S1-HN effectively induced IBV- and NDV-specific antibodies, increased key cytokine levels, and stimulated mucosal immune responses, resulting in reduced viral loads, and alleviated clinical symptoms in vaccinated chickens. These findings indicate that the PAD-S1-HN vaccine provides strong immunogenicity and protective efficacy against IBV and NDV infections. Therefore, it presents a promising alternative to conventional vaccines, offering a novel approach for improving poultry disease management.

**Supplementary Information:**

The online version contains supplementary material available at 10.1186/s13567-025-01528-6.

## Introduction

Infectious bronchitis virus (IBV) is a member of the *Coronaviridae* family and belongs to the genus *Gammacoronavirus* [1]. IBV primarily infects chickens, including broilers, layers, and breeders, causing infectious bronchitis (IB), an acute and highly contagious disease [2]. The IBV genome consists of a single-stranded positive-sense RNA with a length of approximately 27.6–28.3 kilobases [1–3]. The IBV genome encodes multiple structural proteins essential for the virus's life cycle. Its genome is organized as follows: 5'untranslated region (UTR)—ORF1a and ORF1b—spike protein (S protein)—envelope protein (E protein)—membrane protein (M protein)—nucleocapsid protein (N protein)—3'untranslated region (UTR) [4]. This genomic organization allows efficient transcription and replication, as well as the production of various essential proteins for viral assembly and pathogenicity. Owing to its high mutation rate and recombination events [3, 5], IBV exhibits genetic variability, leading to the emergence of various strains and complicating vaccine development and disease management.

The S protein is the most studied structural protein of IBV. It is a trimeric transmembrane protein that can be cleaved into S1 and S2 subunits. S1 contains at least two domains that may be involved in receptor binding and is considered the primary determinant of host and tissue tropism [6]. The S protein includes key antigenic epitopes, including neutralizing epitopes, making it a target for vaccine design and immunotherapy [7]. According to previous studies, on the basis of the complete nucleotide sequence of the S1 gene, IBV can be classified into six genotypes (GI-GVI), with significant differences between these genotypes [8]. However, cross-protection between different IBV genotypes is limited. However, the QX genotype (GI-19) is currently one of the most prevalent strains of IBV in China, especially in southern China, where the detection rate of the QX strain is particularly high, followed by that of GI-1 and GI-13 [9]. Furthermore, the detection rate of the QX (GI-19) strain is also increasing globally [10–12]. The QX (GI-19) strain has been widely used in the poultry industry worldwide, leading to significant economic losses. This strain has spread widely in the poultry industry, causing significant economic losses [13]. Its high transmissibility and variability make control and prevention challenging, and existing vaccines offer limited efficacy against emerging variants, exacerbating the threat to poultry farming.

Newcastle disease virus (NDV) belongs to the family *Paramyxoviridae* and the genus *Orthoavulavirus* [14]. NDV has a single-stranded negative-sense RNA genome that is approximately 15.2 kb in length [15]. The genome is nonsegmented and encodes six major structural proteins, organized in the following order: 3'—nucleocapsid protein (N)—phosphoprotein (P)—matrix protein (M)—fusion protein (F)—hemagglutinin-neuraminidase protein (HN)—large polymerase protein (L)—5'. NDV has a broad host range, infecting mainly poultry, such as chickens, turkeys, and pigeons, but it can also infect wild birds and a few mammals. NDV causes Newcastle disease (ND), a highly contagious and often fatal poultry disease, leading to significant economic losses in the poultry industry [16]. Clinical signs in poultry include respiratory distress, neurological symptoms, and a significant decrease in egg production [17]. The F48E9 strain is a standard strain widely used in NDV research in China and belongs to the IX genotype. It is a highly pathogenic NDV strain [18]. In recent years, genotypes VII and IX of NDV have become highly prevalent in China and across Asia. The LaSota strain (genotype II) is commonly used for vaccination [19]; however, with the genetic evolution of NDV, its compatibility with the currently circulating VII and IX genotype strains has decreased [18]. This reduced compatibility may lead to a decline in immune protection efficacy, and there is a potential risk of vaccine failure. The protein encoded by the HN gene has hemagglutinin activity and neuraminidase activity, making it a key factor for NDV binding to host cell surface receptors and entering cells. As an antigen in vaccines, the HN gene can induce a strong antibody response, primarily providing protection through humoral immunity [20]. On the other hand, the F protein is responsible for the fusion of the virus with the host cell membrane and primarily mediates cellular immune responses, including T-cell responses. Although antibodies against the F protein also have neutralizing effects, their role in immune protection is more pronounced in cellular immunity [21]. In various studies and developments of NDV vaccines, the HN protein has been shown to induce broad immune responses and provide strong protective efficacy [22].

IBV and NDV are two of the most economically significant pathogens in the poultry industry worldwide [23]. The concurrent circulation of these viruses poses a significant challenge to poultry farming, creating an urgent need for effective vaccines that can provide broad and long-lasting protection against both diseases. Traditional IBV and NDV vaccines, including inactivated and live attenuated vaccines, have limited efficacy due to antigenic variation, potential virulence reversion, and incomplete protection against emerging strains [24]. The high mutation rates and genetic diversity of the IBV S1 subunit and NDV HN protein complicate vaccine design, which often requires frequent updates to match circulating strains. Thus, there is an urgent need for innovative vaccine platforms to overcome these challenges and provide comprehensive protection.

Recent advances in recombinant viral vector technologies have opened promising avenues for vaccine development [25, 26]. Owing to their nonreplicating nature and high immunogenicity, chimpanzee adenovirus vectors have emerged as versatile platforms for delivering protective antigens from various pathogens [27, 28]. In the field of vaccine development, various viral vector platforms have been extensively studied and applied, including vaccinia virus, adeno-associated virus (AAV), avian adenovirus, and chimpanzee adenovirus (ChAd). Each of these platforms has unique characteristics and advantages. However, chimp ChAd, as nonreplicating adenoviral vector, offers significant benefits. Owing to its lack of replication ability, it effectively prevents the induction of a strong immune response against the adenovirus vector in the host. This makes ChAd an ideal candidate for delivering protective antigens from various pathogens, particularly in the development of both animal and human vaccines. Furthermore, there are no reports of maternal antibodies against ChAd in poultry, which reduces the risk of immune interference from preexisting immunity—an issue commonly associated with avian adenovirus vector vaccines [27]. A recombinant ChAd vector vaccine expressing IBV S1 and NDV HN proteins represents a novel strategy. By targeting the key antigenic epitopes of both viruses, this bivalent vaccine aims to increase immunogenicity, reduce the need for multiple vaccinations, and provide broad-spectrum protection against multiple viral strains.

The aim of this study was to evaluate the immunogenicity and protective efficacy of a recombinant chimpanzee adenovirus vector vaccine expressing IBV S1 and NDV HN proteins in poultry.

## Materials and methods

### Reagents and materials

The pShuttle-CMV plasmid was used for cloning the S1 gene of IBV and the HN gene of NDV and was synthesized by Sangon Biotech Co., Ltd. (Shanghai). The adenovirus vector was obtained from Chengdu Kanghua Biotechnology Co., Ltd. [27] and has the GenBank accession number AF394196.1. The recombinant AdC68 vectors containing the IBV S1 and NDV HN genes were produced using a similar strategy [27]. Dulbecco’s modified Eagle’s medium (DMEM) was used for cell culture and was purchased from Gibco (Thermo Fisher Scientific, Waltham, MA, USA; Cat. No. 11965–092). Foetal bovine serum (FBS) was obtained from Gibco (Thermo Fisher Scientific; Cat. No. 10270–106). Penicillin‒streptomycin solution was added to the culture media at a concentration of 1% (100 units/mL penicillin, 100 µg/mL streptomycin) and was purchased from Gibco (Thermo Fisher Scientific; Cat. No. 15140–122). Phosphate-buffered saline (PBS, pH 7.4) was purchased from Sigma‒Aldrich (St. Louis, MO, USA; Cat. No. P3813). I-CeuI restriction endonuclease and PI-SceI restriction endonuclease were purchased from New England Biolabs (NEB). The transmission electron microscope (JEM-1400 Flash) used in this study was produced by JEOL Ltd., Japan.

### Cell lines, viruses and animals

HEK293 cells were used for the propagation of recombinant adenoviral vectors. The cell line was purchased from the American Type Culture Collection (ATCC; Manassas, VA, USA; Cat. No. CRL-1573). The cells were maintained in DMEM supplemented with 10% FBS and 1% penicillin‒streptomycin. HD11 cells were used to assess the expression of the chimpanzee adenovirus vector vaccines in chicken-derived cells. The chimpanzee adenovirus PAD-S and empty vector chimpanzee adenovirus PAD were rescued in a previous report and stored at −80 °C [27]. The IBV/QX strain and NDV/F48E9 strain were isolated, identified, and preserved in our laboratory. The QX-NDV bivalent attenuated live vaccine was obtained from Qingdao YiBang Bioengineering Co., Ltd. SPF chickens used for in vivo evaluation of the vaccine candidates were sourced from Beijing Boehringer Ingelheim Vital Biotechnology Co., Ltd. All animal experiments were conducted in compliance with institutional guidelines and were approved by the Institutional Animal Care and Use Committee (IACUC).

### Construction and rescue of recombinant ChAd expressing IBV S1 and NDV HN proteins

The genes encoding IBV S1 and NDV HN were synthesized in tandem, with a 6 × His tag added to the C-terminus. These genes were optimized for both human and chicken species and inserted into the pShuttle-CMV shuttle plasmid (Additional file 1). The pShuttle-CMV plasmid containing the target genes and the pAdC68 adenovirus backbone plasmid were digested with the restriction enzymes I-CeuI and PI-SceI. The digested fragments were then ligated and transformed into Stbl2 *E. coli*. After recombination, the adenovirus genome was linearized using the PacI enzyme, followed by transfection into HEK293 cells using Lipofectamine 3000 (Invitrogen, Cat. No. L3000015). The transfected cells were cultured in DMEM containing 10% fetal bovine serum at 37 °C and 5% CO_2_. Plaques appeared within 7–10 days, viral DNA was extracted, and PCR analysis was performed to confirm the successful recombination of the IBV S1 and NDV HN genes. Successful amplification indicated the preliminary rescue of the virus. The PCR primers used were as follows: upstream primer: tatggcagggaggagtatt; downstream primer: tcgatatctgaccgattcat, with an amplification product size of 4907 bp. The supernatant containing the recombinant adenovirus was collected and subjected to three rounds of plaque purification to ensure clonal purity. The resulting recombinant adenovirus was designated PAD-S1-HN. The adenovirus was further purified by caesium chloride ultracentrifugation following previously described methods.

### Purification and titre determination of the recombinant adenovirus

HEK293 cells were cultured in T175 flasks until they reached approximately 80% confluency. The cells were then infected at a multiplicity of infection (MOI) of 5. When 80–90% of the cells displayed cytopathic effects (CPE), the supernatant containing the virus was collected. The supernatant was centrifuged at 3 000 rpm for 10 min at 4 °C to remove cell debris, and the virus was further concentrated using ultrafiltration equipment. A discontinuous CsCl density gradient was prepared with densities of 1.2 g/mL and 1.4 g/mL using 10 mM Tris–HCl buffer (pH 7.5). The concentrated virus mixture was carefully layered on top of the CsCl gradient in ultracentrifuge tubes. The tubes were balanced and centrifuged at 100 000 × *g* for 2.5 h at 4 °C using an SW41 rotor. After centrifugation, the virus band became visible under light. The lower, denser band, containing the intact adenovirus, was carefully collected using a syringe. The collected fraction was dialyzed overnight at 4 °C in sterile dialysis buffer (10 mM Tris, 100 mM NaCl, 2 mM MgCl₂, 2% sucrose, pH 7.5) to remove the CsCl. The purified virus was aliquoted and stored at −80 °C for future use.

For titre determination, HEK293 cells were seeded into 96-well plates at a density of 10^5^ cells/mL in DMEM containing 2% FBS. The plates were incubated at 37 °C with 5% CO_2_ for 24 h to allow cell attachment. The recombinant adenovirus was serially diluted tenfold in DMEM, with dilutions ranging from 10^–1^ to 10^–20^. In the first 10 columns of the 96-well plate, 100 µL of each virus dilution was added per well, with one dilution per column. The last two columns received 100 µL of 2% DMEM as a negative control. The plates were incubated at 37 °C for 10 days, and the CPE was monitored daily. The number of wells showing CPE at each dilution was recorded, and the virus titre was calculated via the Reed‒Muench method. The results are expressed as the 50% tissue culture infective dose (TCID_50_), which represents the virus concentration that induces CPE in 50% of the wells at a given dilution, reported as the logarithmic dilution at which this occurs.

### Indirect immunofluorescence assay

To confirm the expression of IBV S1 and NDV HN proteins in HEK293 cells infected with PAD-S1-HN, an indirect immunofluorescence assay (IFA) was performed. Briefly, HEK293 cells were seeded in 24-well plates and cultured until they reached approximately 70–80% confluency. The cells were then infected with recombinant adenovirus at an MOI of 10 and incubated for 24 h. After incubation, the cells were fixed with 4% paraformaldehyde at room temperature for 15 min, followed by permeabilization with 0.1% Triton X-100 for 10 min at room temperature. The cells were blocked with PBS containing 5% bovine serum albumin (BSA) for 1 h at room temperature. The mouse anti-His-tag primary antibody, which was diluted 1:2000 in blocking buffer, was subsequently added to the cells, which were subsequently incubated overnight at 4 °C. The cells were washed three times with PBS and incubated with Alexa Fluor 488-conjugated goat anti-mouse secondary antibody (1:500) at room temperature in the dark for 1 h. After washing with PBS, the cells were mounted with DAPI-containing mounting medium. The fluorescence was observed, and images were captured using a fluorescence microscope.

### Western blot analysis

To further confirm the expression of IBV S1 and NDV HN proteins in HEK293 cells, western blot analysis was performed using previously reported PAD and PAD-S as controls. HEK293 cells were infected with recombinant adenovirus at an MOI of 10, and the cells were harvested 24 h post-infection. The cells were lysed using RIPA lysis buffer containing protease inhibitors, and the protein concentration was determined using a BCA protein assay kit. Equal amounts of protein (20 μg) were separated by SDS‒PAGE and transferred to a PVDF membrane. The membrane was blocked with 5% skim milk in TBST (Tris-buffered saline containing 0.1% Tween 20) for 1 h at room temperature. The membrane was incubated overnight at 4 °C with a mouse anti-His-tag primary antibody diluted 1:5000 in blocking buffer. After being washed three times with TBST, the membrane was incubated with an HRP-conjugated goat anti-mouse secondary antibody (1:5000) at room temperature for 1 h. The protein bands were detected by enhanced chemiluminescence (ECL) reagent, and images were captured with a chemiluminescence imaging system.

### Analysis of recombinant adenovirus passaging stability and growth kinetics

To assess the passaging stability of PAD-S1-HN, serial passaging was conducted in HEK293 cells. The recombinant virus was passaged 15 times in HEK293 cells. After each passage, viral DNA was extracted with a commercial DNA extraction kit (QIAamp DNA Mini Kit, Qiagen). The presence and integrity of the inserted IBV S1 and NDV HN genes were confirmed by PCR amplification and Sanger sequencing. The sequencing data were analysed to detect any mutations or deletions in the exogenous gene sequences.

To evaluate the growth kinetics of the recombinant chimpanzee adenovirus, the adenovirus PAD was used as a control. HEK293 cells were infected with PAD-S1-HN or PAD at an MOI of 0.01. The cells and supernatants were collected at specific time points post-infection (0, 12, 24, 48, and 72 h), and viral titres were determined by the TCID_50_ method in HEK293 cells. Growth curves were plotted for both the recombinant and empty adenoviruses to analyse potential differences in replication efficiency and viral dynamics.

### Transmission electron microscopy of purified recombinant ChAd

The purified PAD-S1-HN virus was centrifuged at 100 000 × *g* for 2 h at 4 °C. The viral pellet was carefully resuspended in 50 µL of PBS. A carbon-coated copper grid (200 mesh) was glow-discharged for 30 s to enhance the adsorption of viral particles. Then, 5 µL of the concentrated viral suspension was added to the grid and allowed to adsorb at room temperature for 10 min. The excess viral suspension was carefully removed with filter paper. The grid was then immediately stained with 5 µL of 1% phosphotungstic acid (PTA) solution (pH 7.0) for 30 s. Excess stain was removed with filter paper, and the grid was air-dried for 10 min. The stained grid was observed via transmission electron microscopy (TEM) at an accelerating voltage of 80 kV. Images of the adenovirus particles were captured to assess their morphology and particle integrity.

### Expression of recombinant adenovirus in chicken HD11 cells

To analyse the expression of IBV S1 and NDV HN proteins from the recombinant chimpanzee adenovirus in chicken-derived cells, the HD11 cell line was selected for validation. Briefly, HD11 cells were seeded in 24-well plates and cultured until they reached approximately 70–80% confluency. The cells were then infected with the recombinant adenovirus at an MOI of 10 and incubated for 24 h. After incubation, western blotting and indirect IFA were performed following the procedures described earlier to verify protein expression.

### Immunization

A total of 120 seven-day-old chickens were randomly divided into four groups, with 30 chickens per group (Table 1): (1) the QX + NDV vaccine group, (2) the PAD adenovirus negative control group, (3) the PBS blank control group, and (4) the recombinant chimpanzee adenovirus PAD-S1-HN vaccine group. Immunization was administered via nasal and ocular routes at 7 days of age, with a booster immunization given at 21 days of age. Serum samples were collected from each group on days 7, 14, 21, and 28 post-immunization to assess IBV- and NDV-specific antibody levels, as well as the levels of the cytokines IL-2, IL-4, IL-6, and IFN-γ. The collected serum samples were stored at −20 °C and analysed using enzyme-linked immunosorbent assay (ELISA). Throughout the immunization period, weekly body weight measurements were recorded to assess growth performance, and the data were analysed for significant differences between the groups. Environmental samples were collected weekly from each group to verify the safety of the recombinant adenovirus vaccine. On days 14 and 28 postimmunization, three chickens from each group were randomly selected for euthanasia. Tracheal and intestinal lavage fluid samples were collected to detect IBV- and NDV-specific IgA levels, as well as total mucosal IgA antibodies. Antibody titres in the samples were analysed by ELISA.

**Table 1:** **Groups of chickens immunized with recombinant adenovirus**

Group	Vaccine	Primer dosage	Boost dosage	Challenge	Dose
1	PBS	200 μL	200 μL	QX + F48E9	30
2	PAD	10^10^ TCID_50_	10^10^ TCID_50_	QX + F48E9	30
3	PAD-S1-HN	10^10^ TCID_50_	10^10^ TCID_50_	QX + F48E9	30
4	QX + NDV	200 μL	200 μL	QX + F48E9	30

### ELISA and haemagglutination inhibition

Commercial ELISA kits from Jiangsu Meimian Industrial Co., Ltd., China, were used to measure IBV-specific antibodies, cytokines (IL-2, IL-4, IL-6, and IFN-γ), chicken-specific IgA antibodies and total mucosal IgA antibodies. were used according to the manufacturer’s instructions. Blood samples were collected from all groups on days 7, 14, 21, and 28 post-immunization. After collection, the blood samples were centrifuged at 3000 rpm for 10 min to separate the serum, which was then stored at −20 °C for later analysis.

For IBV-specific antibodies, 100 µL of each diluted serum sample was added to each well of a 96-well plate precoated with IBV antigen. The plates were incubated at 37 °C for 1 h. After the plates were washed with PBST, 100 µL of HRP-conjugated secondary antibody was added to each well and incubated for 30 min at 37 °C. Following another wash, 100 µL of TMB substrate solution was added for color development, and the reaction was stopped with 50 µL of stop solution. The optical density (OD) values were measured at 450 nm using a microplate reader.

Cytokine levels (IL-2, IL-4, IL-6, and IFN-γ) in the serum samples were measured using specific ELISA kits following a protocol similar to that used for IBV-specific antibody detection. The ELISA kits provided specific antibodies and standards, and the results were quantified on the basis of standard curves. Commercial ELISA kits (Jiangsu Meimian Industrial Co., Ltd., China) were used to detect IBV- and NDV-specific IgA antibodies, as well as total mucosal IgA antibodies, in tracheal and intestinal lavage fluid. The lavage fluid samples were appropriately diluted, and the ELISA procedure followed the manufacturer’s instructions, similar to the serum sample analysis.

To assess the hemagglutination inhibition (HI) titres of HN-specific antibodies, HI tests were performed. Serum samples were collected as described above and serially diluted twofold in PBS. A standardized amount of NDV antigen (4 HA units, NDV/F48E9 strain) was mixed with the diluted serum and incubated for 30 min at room temperature. Then, 1% chicken red blood cells (RBCs) were added, and the mixture was incubated for an additional 30 min. The highest serum dilution that completely inhibited hemagglutination was recorded as the HI titre.

### Challenge experiment

At 35 days of age, the chickens were subjected to a challenge infection using a mixed viral suspension containing 10^5.4^ EID_50_ of the IBV/QX strain and 10^4.5^ EID_50_ of the NDV/F48E9 strain (Table 1). Each chicken received 0.1 mL of the viral mixture intranasally, with 0.05 mL administered per nostril. The inoculum was carefully controlled using a pipette to ensure precise dosing, and the challenge experiment was conducted under standard biosafety conditions.

On days 2, 5, and 8 post-challenge (dpc), throat swabs and cloacal swabs were collected from 5 randomly selected chickens per group, with three replicates for each sample. These swabs were placed in virus transport medium and stored at −80 °C until further analysis. Viral shedding was assessed by RT‒qPCR. Daily monitoring of the chickens included clinical observations such as feed intake, water consumption, disease symptoms, and mortality. Any unusual behaviors or symptoms were documented, and necropsies were performed on chickens that died during the challenge to determine the cause of death.

On day 10 dpc, all remaining chickens were euthanized. Three randomly selected chickens were sampled from each group, with three replicates for each sample. Tissue samples from the trachea, kidneys, lungs, proventriculus, and duodenum were collected and analysed for viral load by RT‒qPCR. Additionally, tissue sections from the trachea, kidneys, lungs, and spleen were taken for histopathological examination to observe pathological changes associated with the challenge infection.

### RT‒qPCR

To quantify IBV and NDV viral loads, total RNA was extracted from collected throat swabs, cloacal swabs, and tissue samples (trachea, kidneys, lungs, proventriculus, and duodenum) using TRIzol reagent (Thermo Fisher Scientific) according to the manufacturer’s instructions. The concentration and purity of the RNA were measured using a NanoDrop spectrophotometer (Thermo Fisher Scientific). For each reaction, 1 μg of RNA was reverse transcribed into cDNA using a cDNA reverse transcription kit (Thermo Fisher Scientific).

Subsequent RT‒qPCR was performed in triplicate, and the average Ct values were used for analysis. RT‒qPCR was conducted using the QuantStudio 3 Real-Time PCR System (Applied Biosystems, USA) with SYBR Green qPCR Master Mix (Applied Biosystems, USA). Each 20 μL reaction mixture contained 2 μL of cDNA, 10 μL of TaqMan Fast Advanced Master Mix, 0.5 μL each of forward and reverse primers (10 μM), 0.5 μL of the probe (5 μM), and nuclease-free water.

The thermal cycling conditions were 95 °C for 20 s, followed by 40 cycles of 95 °C for 3 s and 60 °C for 30 s. The primers used to detect the IBV/QX strains were as follows:

- **QX-F:** CGCTCAAGTTCAAGACCTGCTA
- **QX-R:** CATCATCCTGCTTCTTCGGCTT

The standard curve equation for IBV detection was y = − 3.451x + 35.787y = −3.451x + 35.787y =  − 3.451x + 35.787, with an efficiency of E = 0.949E = 0.949E = 0.949 and R2 = 0.999R^2 = 0.999R2 = 0.999.

The primers used to detect the NDV/F48E9 strain were as follows:

- **NDV-F:** ATGGGCYCCAGAYCTTCT
- **NDV-R:** CTGCCACTGCTAGTTGTGATAAT

The standard curve equation for NDV detection was y = − 3.440x + 35.731y = −3.440x + 35.731y =  − 3.440x + 35.731, with an efficiency of E = 0.953E = 0.953E = 0.953 and R2 = 0.999R^2 = 0.999R2 = 0.999 (Additional file 3).

### Haematoxylin & eosin (HE) staining

Tissue samples from the trachea, kidneys, lungs, and spleen were collected and immediately fixed in 4% neutral buffered formalin for 24–48 h to preserve the cellular and tissue structure. The samples were then sent to Chengdu Seville Biotechnology Co., Ltd., for HE staining. The stained sections were observed under an optical microscope to assess pathological changes in the tissues.

### Data analysis

Statistical analysis was performed with GraphPad Prism 9.0 (GraphPad Software, Inc., USA). The data were analysed by one-way ANOVA or Student’s *t* test to assess the significance of differences between experimental groups. A *p* value of less than 0.05 was considered statistically significant (*), whereas a *p* value of less than 0.01 was considered highly significant (**). All the results are presented as the means ± standard errors of the means (SEM).

## Results

### Characterization of recombinant ChAd expressing IBV S1 and NDV HN proteins

**Figure 1:**
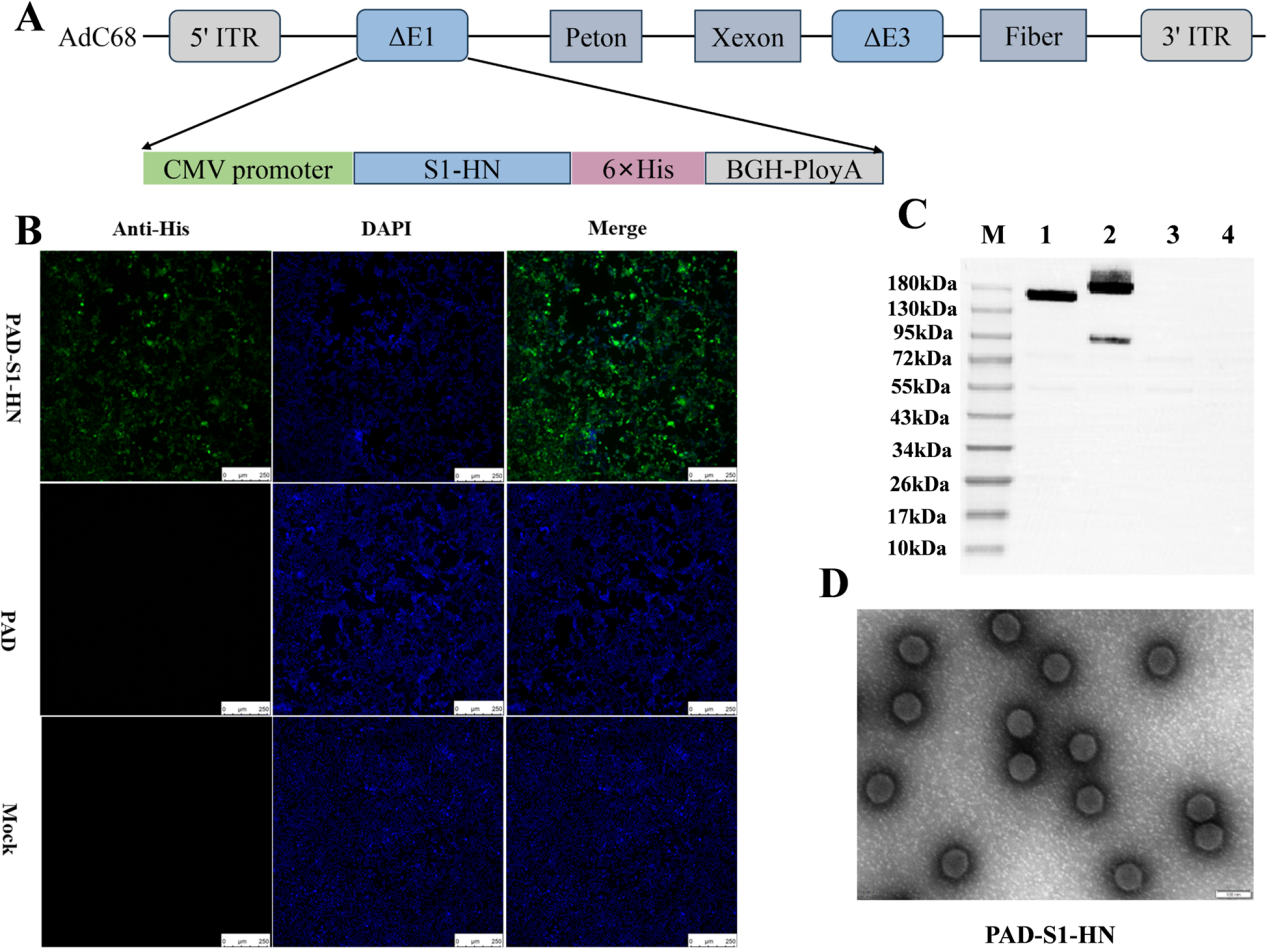
**Rescue and characterization of the recombinant chimpanzee adenovirus PAD-S1-HN. A** Schematic diagram of the construction of the recombinant chimpanzee adenovirus vector. **B** IFA identification of the expression of the target protein PAD-S1-HN from the recombinant chimpanzee adenovirus. **C** Western blot analysis of target protein expression of the recombinant chimpanzee adenovirus PAD-S1-HN, PAD-S1-HN (Lane 1), the control recombinant adenovirus PAD-S from a previous study (Lane 2), the empty adenovirus PAD (Lane 3), and the normal cell control (Lane 4). **D** Transmission electron microscopy of the recombinant adenovirus (150 000 ×).

As shown in the schematic diagram in Figure 1A, recombinant chimpanzee adenovirus expressing IBV S1 and NDV HN proteins was successfully rescued in HEK293 cells with a ChAd vector. Viral DNA was extracted from cells showing CPE, and PCR analysis confirmed the presence of the IBV S1 and NDV HN genes, confirming their successful integration into the adenovirus genome (data not shown). The purified recombinant adenovirus was observed by TEM. The virus particles exhibited typical adenovirus morphology, with an icosahedral capsid structure approximately 80–100 nm in diameter. The morphology and size were consistent with those of the previously described recombinant chimpanzee adenovirus PAD-S and the empty adenovirus PAD, confirming the successful production and purification of the recombinant virus (Figure 1D).

Following 24 h of infection with recombinant adenovirus, the expression of IBV S1 and NDV HN proteins in HEK293 cells was confirmed by IFA and western blot. The IFA results revealed a stronger fluorescent signal in HEK293 cells infected with PAD-S1-HN than in those infected with the adenovirus PAD, indicating successful expression of the target proteins (Figure 1B). Additionally, western blot analysis revealed bands corresponding to the IBV S1 and NDV HN fusion proteins at approximately 160 kDa (Figure 1C, Lane 1), further confirming their expression. The previously studied recombinant adenovirus PAD-S was used as a positive control, showing specific bands at 180 kDa (S) and 84 kDa (S2) (Figure 1C, Lane 2), while the empty adenovirus PAD served as a negative control (Figure 1C, Lane 3), and normal cells were used as a blank control (Figure 1C, Lane 4), with no specific bands detected.

The same conditions were applied to HD11 cells to evaluate the expression of foreign proteins in chicken-derived HD11 cells. The IFA and WB results were consistent with those observed in HEK293 cells (Additional file 4), further validating the successful expression of the target proteins by the recombinant adenovirus.

### Genetic stability and growth kinetics analysis of the recombinant adenovirus

The genetic stability of the recombinant ChAd expressing IBV S1 and NDV HN proteins was evaluated through serial passaging in HEK293 cells. The virus was passaged 15 times, and viral DNA was extracted for sequencing analysis after passages 3, 6, 9, 12, and 15. The sequences of the IBV S1 and NDV HN genes remained unchanged throughout all passages (data not shown), confirming the genetic stability of the recombinant adenovirus during multiple rounds of passaging.

**Figure 2:**
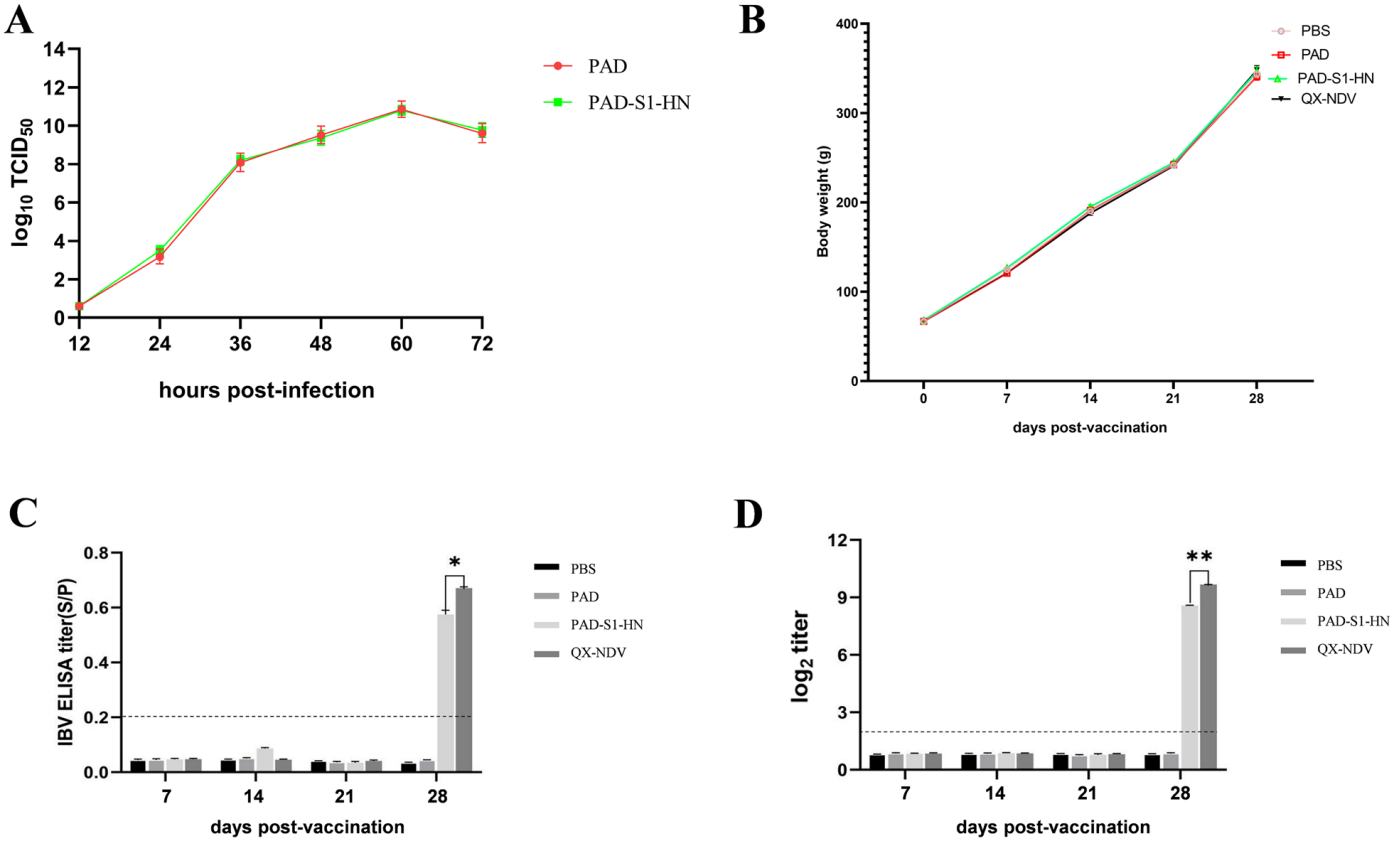
**Growth kinetics and preliminary immunogenicity investigation of the recombinant chimpanzee adenovirus PAD-S1-HN in chickens. A** One-step growth curve of the recombinant adenovirus PAD-S1-HN, with the empty adenovirus PAD used as a control. **B** Growth trends of chicken body weight after immunization in different groups. **C** IBV-specific IgG antibody levels in chicken serum. **D** NDV-specific IgG antibody levels in chicken serum.

The titre of the purified recombinant adenovirus PAD-S1-HN was 10^10.39^ TCID_50_, while the previously described adenovirus PAD was used as a control, with a titre of 10^10.22^ TCID_50_. To analyse the growth kinetics of the recombinant ChAd, HEK293 cells were infected with the virus at an MOI of 0.01. The viral titres in HEK293 cells were measured at different time points post-infection. The results revealed that the growth kinetics of the recombinant virus were similar to those of the control empty chimpanzee adenovirus (Figure 2A). Both viruses reached peak titres at 60 h post-infection, indicating that the insertion of the IBV S1 and NDV HN genes did not adversely affect the replication efficiency of the recombinant virus.

### Immunogenicity of the recombinant ChAd in chickens

Throughout the immunization period, chickens from all the experimental groups were monitored weekly for body weight, behavior, and overall health status. Environmental samples were collected weekly to verify whether there was any risk of viral shedding from the recombinant adenovirus vaccine. The results revealed that all chickens exhibited normal behavior, with no signs of discomfort or disease. The average body weight of the chickens in all the groups steadily increased during the experiment, indicating normal growth patterns (Figure 2B), without any negative impact from the immunization regimen. Moreover, adenovirus was not detected in chicken feed, drinking water, or feces (Additional file 4). Necropsies conducted on randomly selected chickens euthanized at 14 and 28 days post-vaccination (dpv) revealed normal organ structures. Overall, the recombinant adenovirus demonstrated good safety in chickens.

Specific antibodies against IBV and NDV were measured using ELISA and HI assays. At 28 dpv, both the recombinant adenovirus PAD-S1-HN group and the commercial vaccine QX-NDV group were positive for specific antibodies against IBV and NDV (Figure 2C, D), whereas the PAD and PBS control groups tested negative. These results indicate that in the early stages of vaccination, antibody levels were insufficient to be detected, but booster immunization at 21 days significantly increased antibody levels. These findings suggest that the recombinant adenovirus PAD-S1-HN can elicit specific antibodies against IBV and NDV in chickens and that booster immunization is necessary.

**Figure 3:**
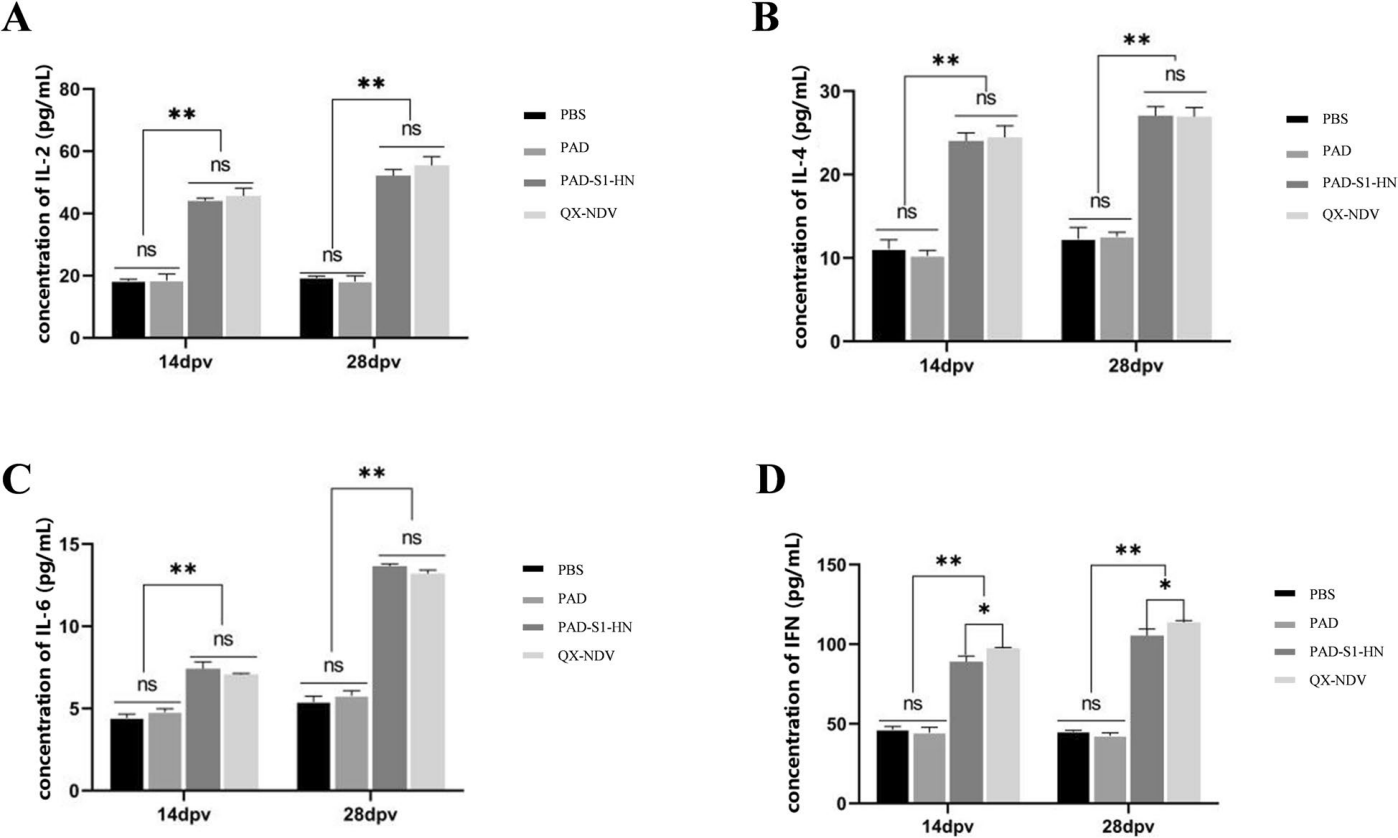
**Measurement of major cytokine levels in chickens post-immunization. A** Measurement of IL-2 levels in chickens at 14 and 28 days after the first immunization (dpv: days post-vaccination). **B** Measurement of IL-4 levels in chickens at 14 and 28 days after the first immunization. **C** Measurement of IL-6 levels in chickens at 14 and 28 days after the first immunization. **D** Measurement of IFN-γ levels in chickens at 14 and 28 days after the first immunization.

After the levels of the NDV- and IBV-specific antibodies were measured, the levels of the cytokines IL-2, IL-4, IL-6, and IFN-γ were also evaluated (Figure 3). These cytokines are key indicators of T-cell function and can provide insights into the nature of the immune response induced by a vaccine. IL-2 and IFN-γ are associated primarily with Th1 cells, which play critical roles in mediating cellular immune responses, whereas IL-4 and IL-6 are linked to Th2 cells, which drive humoral immunity. In this study, at both 14 dpv and 28 dpv, the levels of IL-2, IL-4, and IL-6 in the PAD-S1-HN group and the commercial QX-NDV group were significantly greater (*p* < 0.01) than those in the PAD and PBS control groups were (Figure 3A, B, C). Similarly, the levels of IFN-γ were significantly greater (*p* < 0.01) in both the PAD-S1-HN and QX-NDV groups than in the control group. However, the QX-NDV vaccine group presented significantly higher IFN-γ levels than did the PAD-S1-HN group (Figure 3D). This might be attributed to the continuous low-level viral replication from the live attenuated vaccine, which could lead to stronger immune stimulation, particularly in activating a Th1-type immune response, resulting in increased IFN-γ production. Interestingly, no significant difference in cytokine levels was observed between the PBS and PAD groups, possibly because the empty chimpanzee adenovirus lacks the ability to replicate, leading to lower immunogenicity, which is also an advantage as a vaccine vector.

**Figure 4:**
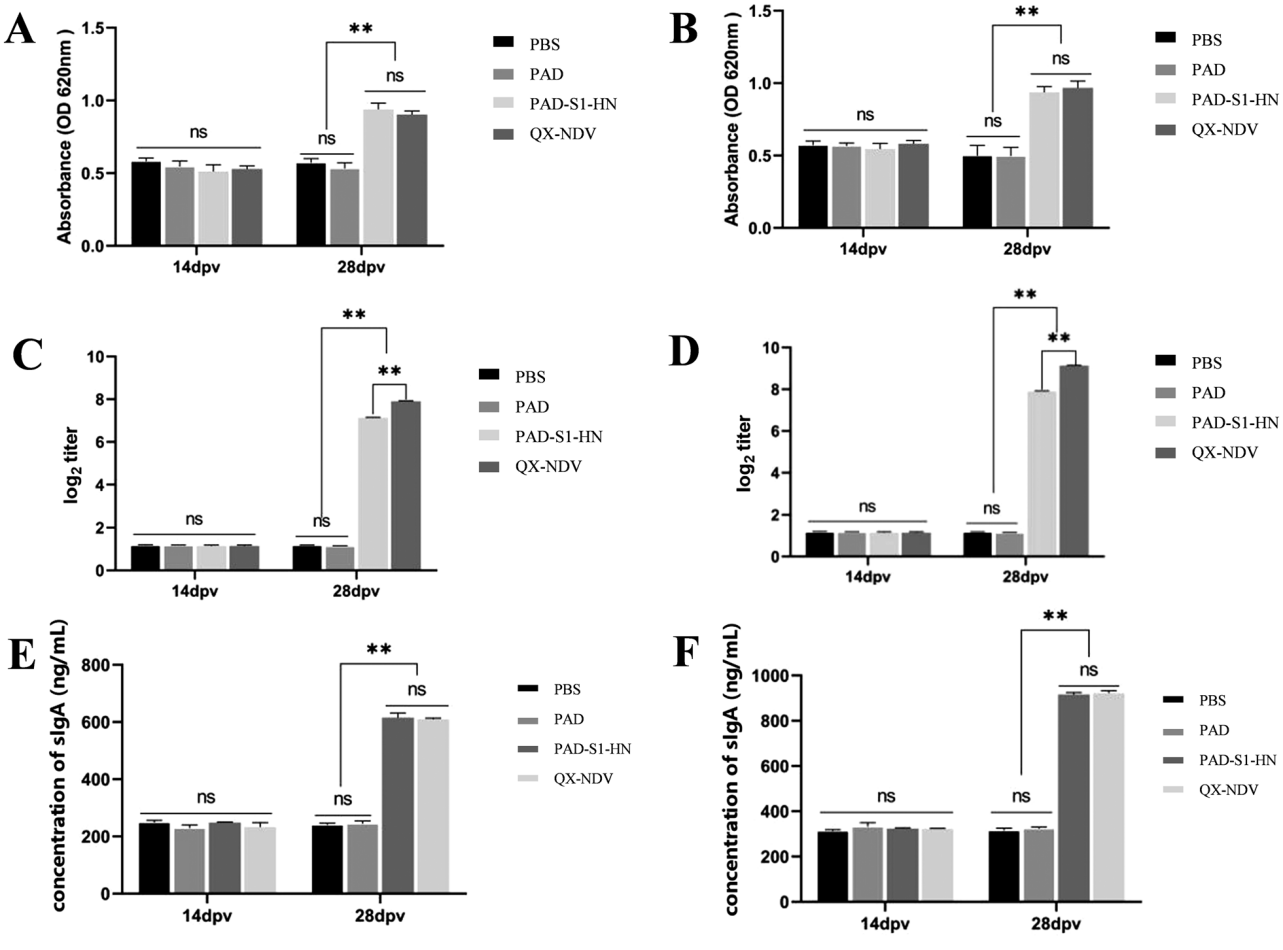
**Detection of specific mucosal IgA antibodies and total mucosal IgA antibodies. A** IBV-specific IgA results in chicken trachea at 14 and 28 days after the first immunization. **B** IBV-specific IgA results in chicken intestines at 14 and 28 days after the first immunization. **C** NDV-specific IgA results in chicken trachea at 14 and 28 days after the first immunization. **D** NDV-specific IgA results in chicken intestines at 14 and 28 days after the first immunization. **E** Total mucosal IgA results in chicken trachea at 14 and 28 days after the first immunization. **F** Total mucosal IgA results in chicken intestines at 14 and 28 days after the first immunization.

Tracheal and intestinal lavage fluid samples were collected, and IBV- and NDV-specific IgA levels, as well as total mucosal IgA antibody levels, were measured by ELISA (Figure 4). The results revealed that at 14 dpv, there was no significant difference in IBV-specific IgA levels in the trachea (Figure 4A) or intestines (Figure 4B) among the groups. At 28 dpv, IBV-specific IgA levels in both the trachea (Figure 4A) and intestines (Figure 4B) were greater in the QX-NDV vaccine group than in the PAD-S1-HN group, with no significant difference between them, but they were significantly greater than those in the PAD and PBS groups (*p* < 0.01). With respect to NDV, at 14 dpv, there was no significant difference in IgA levels in the trachea or intestines across the groups. However, at 28 dpv, both the QX-NDV vaccine group and the PAD-S1-HN group effectively induced NDV-specific IgA, with significantly higher levels than the PAD and PBS groups did (*p* < 0.01), but the QX-NDV vaccine group had significantly higher IgA levels than the PAD-S1-HN group did (*p* < 0.01) (Figure 4C, D). The results for total mucosal IgA in the tracheal lavage (Figure 4E) and intestinal lavage (Figure 4F) showed no significant differences among the groups at 14 dpv. At 28 dpv, the IgA concentrations in the PAD-S1-HN and QX-NDV vaccine groups were greater, with no significant differences between them, but were significantly greater than those in the non-vaccinated groups (*p* < 0.01).

In summary, the recombinant chimpanzee adenovirus vaccine PAD-S1-HN can induce a strong immune response in chickens.

### Protective efficacy of recombinant ChAd against IBV and NDV challenge

**Figure 5:**
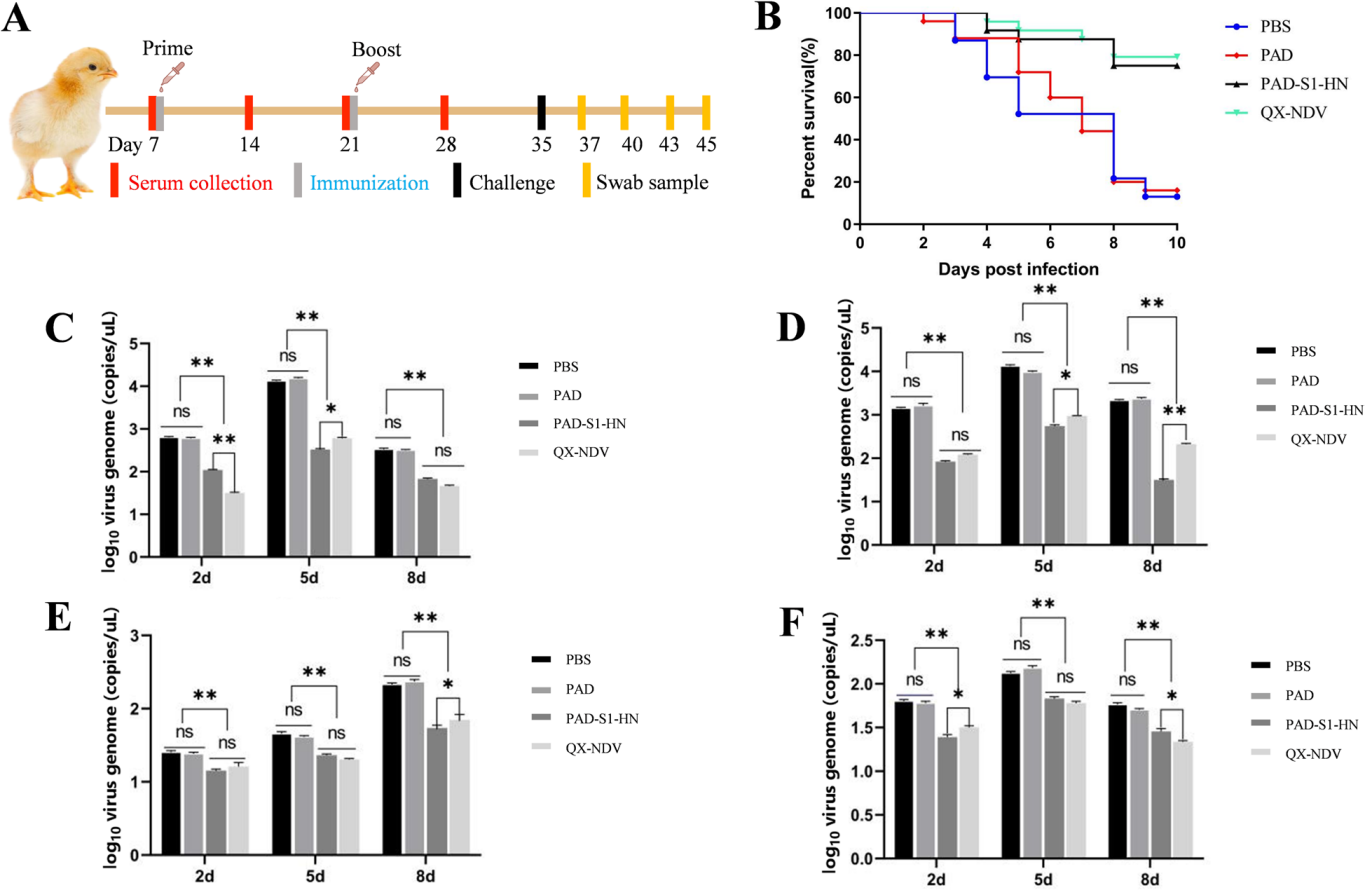
**Viral challenge in chickens. A** Schematic diagram of the animal experimental protocol. **B** Survival curve after IBV and NDV challenge (*n* = 24). **C** Detection of IBV shedding in throat swabs on days 2, 5, and 8 post-challenge. **D** Detection of IBV shedding in cloacal swabs on days 2, 5, and 8 post-challenge. **E** Detection of NDV shedding in throat swabs on days 2, 5, and 8 post-challenge. **F** Detection of NDV shedding in cloacal swabs on days 2, 5, and 8 post-challenge.

The immunization and viral challenge schedule for the chickens is shown in Figure 5A. After challenge with IBV/QX and NDV/F48E9, chickens in the non-vaccinated groups (PAD and PBS) started showing typical clinical symptoms such as head shaking, sneezing, and poor mental state on the second day, followed by feather ruffling, clustering, wet droppings, and reduced food and water intake, along with noticeable conjunctivitis. In contrast, only a few chickens in the PAD-S1-HN and QX-NDV vaccine groups presented mild clinical symptoms at a later stage.

Within 10 dpc, the morbidity rates in the PBS and PAD groups were 91.6% (22/24) and 87.5% (21/24), respectively. In contrast, the morbidity rates for the PAD-S1-HN and QX-NDV vaccine groups were significantly lower at 29.1% (7/24) and 25% (6/24), respectively, with milder symptoms. Mortality rates also varied across the groups. On the second day after challenge, chickens started to die, and by day 10 post-challenge, survival rates were significantly higher in the vaccinated groups. The survival rates were 75% (18/24) in the PAD-S1-HN group and 79% (19/24) in the QX-NDV live-attenuated vaccine group, whereas the survival rates were low in the non-vaccinated groups, with 16.6% (4/24) in the PAD group and 12.5% (3/24) in the PBS group (Figure 5B).

Quantitative analysis of viral shedding was performed on throat and cloacal swabs collected on days 2, 5, and 8 post-challenge by the established RT‒qPCR method for NDV and IBV. Five chickens were randomly selected from each group for sampling, with triplicate testing for each sample. For IBV, viral shedding was detected in the throat swabs (Figure 5C) and cloacal swabs (Figure 5D) of all the groups on days 2, 5, and 8 post-challenge. However, viral gene levels were significantly lower in the vaccinated groups (PAD-S1-HN and QX-NDV) than in the nonvaccinated groups (PBS and PAD) (*p* < 0.01). No significant differences were observed between the PBS and PAD groups. Viral loads peaked on day 5 post-challenge in both throat and cloacal swabs, but at this peak, the viral load in the PAD-S1-HN group was significantly lower than that in the QX-NDV group (*p* < 0.05). On day 8 post-challenge, the IBV viral loads in throat swabs were similar between the PAD-S1-HN and QX-NDV groups, but the cloacal swabs from the PAD-S1-HN group presented significantly lower viral loads than did those from the QX-NDV group (*p* < 0.01). Overall, compared with the nonvaccine control groups, the PAD-S1-HN group presented significantly reduced IBV shedding and generally lower viral loads than the QX-NDV group did, indicating the effectiveness of the recombinant adenovirus vaccine in reducing IBV shedding and potential infection risk.

For NDV detection, the viral load in throat swabs (Figure 5E) increased steadily across all groups, with the highest viral load observed on day 8 post-challenge. Compared with the nonvaccine groups (PBS and PAD), the vaccinated groups (PAD-S1-HN and QX-NDV) presented significantly lower viral loads, and the PAD-S1-HN group presented significantly lower viral loads than did the QX-NDV group. In cloacal swabs (Figure 5F), the viral load peaked on day 5 post-challenge in all groups. At this point, the viral loads in the PAD-S1-HN and QX-NDV groups were significantly lower than those in the nonvaccine groups. However, no significant difference was observed between the PAD-S1-HN and QX-NDV groups. On day 8 post-challenge, the NDV viral loads in the PAD-S1-HN group remained significantly lower than those in the PBS and PAD groups but were greater than those in the QX-NDV group. In summary, the recombinant adenovirus vaccine PAD-S1-HN significantly reduced viral shedding during IBV and NDV infection, providing protection comparable to that of the commercial QX-NDV live-attenuated vaccine. These findings suggest that the PAD-S1-HN vaccine has potential as an alternative immunization strategy.

**Figure 6:**
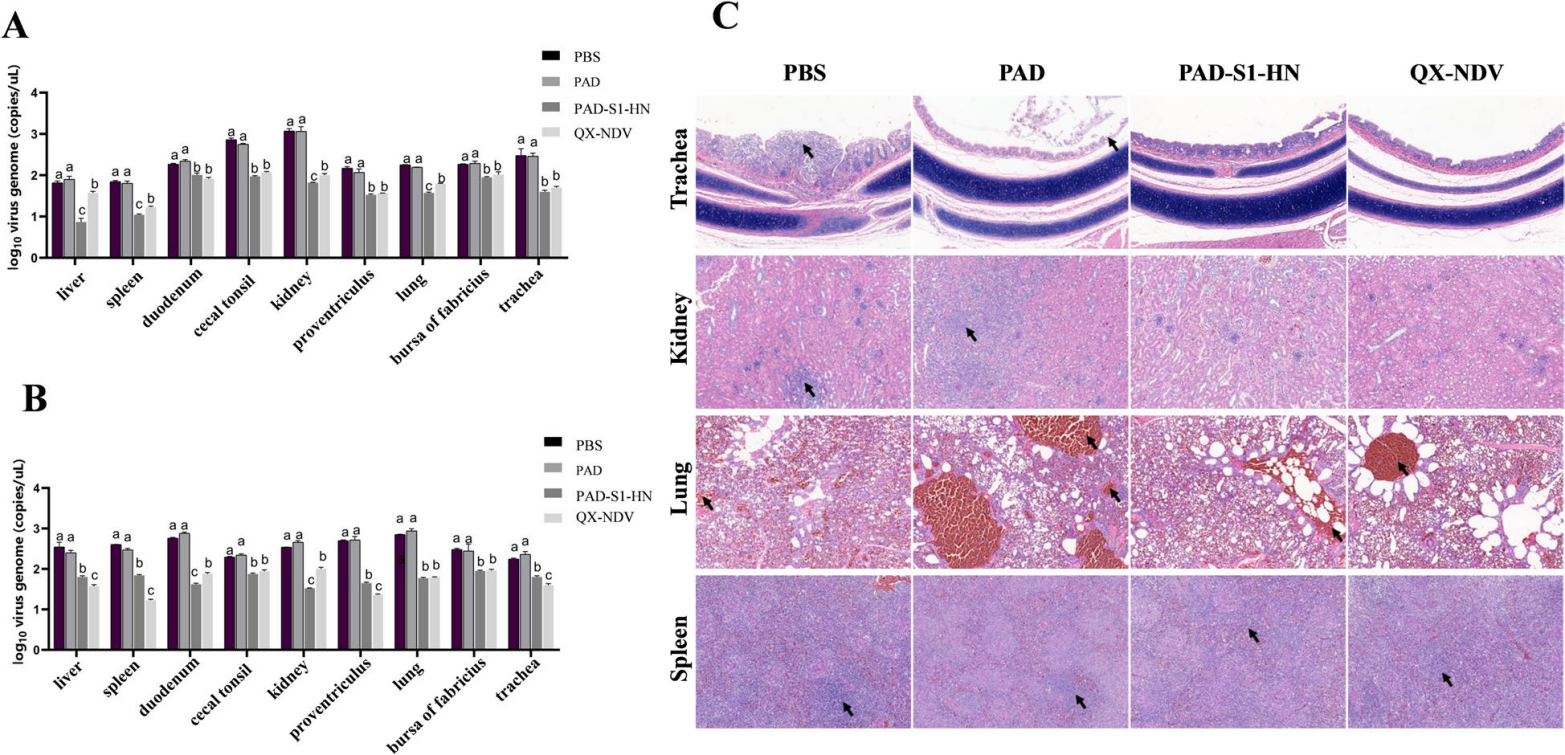
**Measurement of the viral load in organs and pathological analysis post-challenge. A** Viral load of IBV in different organs post-challenge. **B** Viral load of NDV in different organs post-challenge. **C** Pathological changes in major organs and tissues post-challenge.

On day 10 post-challenge, virus quantification was performed on all collected tissues. For IBV (Figure 6A), the highest viral loads were detected in the kidneys, followed by the cecal tonsils, trachea, and bursa of Fabricius, in the nonvaccine groups (PBS and PAD), with no significant differences in viral loads between these two groups. In contrast, the viral loads in all the tissues from the vaccinated groups (PAD-S1-HN and QX-NDV) were significantly lower than those in the nonvaccine groups (*p* < 0.05). In the duodenum, proventriculus, bursa of Fabricius, and trachea, there was no significant difference in IBV viral loads between the PAD-S1-HN and QX-NDV groups, but the viral loads in the liver, spleen, kidneys, and lungs were significantly lower in the PAD-S1-HN group than in the QX-NDV group.

For NDV (Figure 6B), after challenge with NDV/F48E9, the viral loads in the vaccinated groups (PAD-S1-HN and QX-NDV) were significantly lower than those in the nonvaccine groups (PBS and PAD) (*p* < 0.05). Among the nonvaccine groups, the highest viral loads were detected in the lungs, followed by the duodenum, proventriculus, and other organs, with no significant differences between the PBS and PAD groups. In some tissues, such as the liver, spleen, proventriculus, and trachea, the NDV viral loads in the QX-NDV vaccine group were significantly lower than those in the PAD-S1-HN group (*p* < 0.05). However, in the caecal tonsils, lungs, and bursa of Fabricius, there were no significant differences in viral loads between the PAD-S1-HN and QX-NDV groups. In the kidneys and duodenum, the NDV viral loads in the PAD-S1-HN group were significantly lower than those in the QX-NDV group (*p* < 0.05). Overall, the PAD-S1-HN vaccine provided comparable protection to the commercial QX-NDV vaccine against IBV and NDV infection.

### Histopathological analysis of chicken tissues post-challenge

Following IBV and NDV challenge, common tissue damage induced by the viruses was observed in histopathological sections (Figure 6C). The PBS group presented severe kidney lesions, characterized by noticeable tubular necrosis and inflammatory cell infiltration, whereas the PAD group also presented tubular necrosis. Both the PAD and PBS groups exhibited necrosis of the tracheal mucosal lamina propria accompanied by inflammatory cell infiltration; however, no pathological changes were observed in the trachea or kidneys of the PAD-S1-HN and QX-NDV groups. Severe splenic nodule atrophy and lymphocyte depletion were present in the PAD and PBS groups, whereas only mild splenic nodule atrophy was observed in the PAD-S1-HN and QX-NDV groups. Additionally, the lungs of the PAD and PBS groups presented severe congestion and haemorrhage, whereas those of the PAD-S1-HN and QX-NDV vaccine groups presented only mild haemorrhage. The details of the organ pathological changes are listed in Additional file 2. Overall, the PAD-S1-HN and QX-NDV groups presented milder histopathological changes, with no significant differences between them, and the severity of these changes was significantly lower than that of the PAD and PBS groups. These results further support that PAD-S1-HN provides the same level of protection as the commercial vaccine for chickens.

## Discussion

The poultry industry plays a crucial role in global food security, with chicken meat and eggs serving as essential sources of high-quality protein for billions of people [29]. However, the health and productivity of poultry are constantly threatened by infectious diseases, with IBV and NDV being among the most severe [30]. These viruses cause a reduction in egg production, poor meat quality, and increased mortality, leading to significant economic losses. Despite the widespread use of current vaccines, controlling IBV and NDV remains challenging because of incomplete protection and the emergence of new viral strains. Therefore, new vaccines that are both effective and adaptable to the evolution of these pathogens are urgently needed. For NDV, the F48E9 strain is a widely used standard virulent vaccine strain in China, whereas the LaSota strain is the most commonly used attenuated vaccine strain globally [18]. The HN protein of the F48E9 strain exhibits stronger immunogenicity and is more effective in stimulating immune responses, particularly in inducing higher levels of antibody production post-vaccination. As a result, it provides stronger protection for poultry. Furthermore, the F48E9 strain has greater antigenic similarity to the circulating NDV strains in China, offering enhanced immune protection against these field strains. The QX genotype (GI-19) is one of the most prevalent strains of IBV in China [31].

Nonreplicating chimpanzee adenoviruses (ChAds) lack essential replication genes, preventing autonomous replication in host cells [32]. The short duration of antigen exposure from these vectors reduces the chances of generating neutralizing antibodies against the vector itself. This allows for multiple vaccinations without preexisting immunity, significantly diminishing the vaccine's efficacy. Additionally, nonreplicating chimpanzee adenovirus vectors can carry large exogenous gene inserts and stably express them in host cells, which fulfils the requirements for complex vaccines or gene therapies [33]. This flexibility enables the design of multiantigen vaccines, opening new possibilities for broader protection against pathogens.

Currently, chimpanzee adenovirus vaccines have not been widely applied in livestock and poultry farming. However, given their numerous advantages, they have potential for controlling IBV and NDV. In this study, we successfully constructed and characterized a recombinant chimpanzee adenovirus expressing both the IBV S1 protein and the NDV HN protein. The immunization results demonstrated that the recombinant vaccine elicited a strong immune response in chickens, including the production of specific antibodies, cytokines, and mucosal immunity, all of which are critical for defending against respiratory pathogens. These findings suggest that the recombinant chimpanzee adenovirus vaccine can serve as a promising alternative for controlling IBV and NDV in poultry, providing robust immune protection and adaptability to viral evolution.

In this study, we successfully developed a recombinant chimpanzee adenovirus-based bivalent vaccine, PAD-S1-HN, capable of providing chickens with protection comparable to that of commercial vaccines against IBV and NDV. This vaccine has the potential to become a new preventive tool for controlling these major poultry diseases.

PAD-S1-HN efficiently expresses both the IBV S1 protein and the NDV HN protein and maintains typical adenovirus morphology and similar growth kinetics. Importantly, the integrity and functional expression of the foreign genes of the vaccine were retained after multiple passages, ensuring its consistency and control during production. In animal studies, the observed protective efficacy of the vaccine was attributed to its strong immunogenicity. The vaccinated chickens produced high titres of IBV- and NDV-specific antibodies, along with a significant increase in the levels of key cytokines such as IL-2, IL-4, IL-6, and IFN-γ. IL-2 and IFN-γ are associated with Th1-type immunity and play key roles in cellular immune responses [34]. The presence of IL-2 indicates T-cell proliferation and activation, whereas IFN-γ enhances macrophage activation, promoting NK cell and cytotoxic T lymphocyte (CTL) activity and reflecting a strong Th1-mediated response. On the other hand, IL-4 and IL-6 are linked to Th2 responses, which drive humoral immunity [35]. IL-4 facilitates the differentiation of naïve T cells into Th2 cells and promotes B-cell proliferation and antibody production, while IL-6 supports B-cell differentiation into plasma cells and has proinflammatory effects.

Moreover, the vaccine significantly stimulated mucosal immunity, as evidenced by the presence of specific IgA antibodies in tracheal and intestinal lavage fluid, which are essential for defending against respiratory and gastrointestinal infections. Interestingly, IFN-γ levels were significantly lower in the PAD-S1-HN group than in the QX-NDV group, possibly due to the stronger immunogenicity of live vaccines, which more closely mimics natural infections and triggers a robust Th1 response. In contrast, recombinant adenovirus vaccines may mainly induce antigen-specific immune responses, with relatively lower immunogenicity in terms of IFN-γ production. Overall, the PAD-S1-HN vaccine not only induced potent systemic and mucosal immune responses but also significantly elevated IBV- and NDV-specific antibody levels and cytokine expression.

In the challenge experiment, the chickens vaccinated with PAD-S1-HN showed strong protection against both IBV and NDV. The vaccine effectively reduced the clinical symptoms, tissue damage, and mortality associated with IBV and NDV infections. Compared with those in the nonvaccinated control groups, the viral loads in various organs of the vaccinated chickens were significantly lower, providing protection comparable to that of commercial vaccines. These findings highlight the potential of the PAD-S1-HN vaccine to induce robust protective immunity in poultry, which is critical for mitigating the impact of these viruses.

However, the vaccine developed in this study has certain limitations, particularly regarding its protective efficacy against other genotypes of IBV and NDV, which remains unclear. Our current research only evaluated the vaccine against specific strains of IBV and NDV, and it is not yet known whether the vaccine can effectively address other genotypes of these viruses. Additionally, for NDV, the F gene plays a crucial role in its pathogenicity. It is involved not only in the fusion of the virus with the host cell membrane but also in the induction of specific cellular immune responses, which are essential for clearing viral infections [21]. Therefore, while the use of the HN protein alone can elicit a strong humoral immune response, it does not fully leverage the advantages of the F protein in mediating cellular immunity. Future studies could consider coexpressing both the HN and F proteins of NDV, as this may increase the diversity of the immune response and provide more comprehensive protection. Furthermore, long-term studies are needed to assess the duration of immune protection associated with the PAD-S1-HN vaccine. Future research should focus on optimizing the vaccine formulation, exploring different administration routes, and conducting large-scale trials to establish its practical application in commercial poultry environments.

In conclusion, this study represents the first successful development of a chimpanzee adenovirus-based bivalent recombinant vaccine for IBV and NDV, providing protection comparable to that of commercial vaccines. This vaccine holds promise as a new tool for preventing IBV and NDV infections in poultry.

## Supplementary Information

**Additional file 1. Optimized Sequences of IBV S1 and NDV HN Proteins.**

**Additional file 2. Pathological Changes and Scoring of Organs Post IBV and NDV Challenge.**

**Additional file 3. Establishment of SYBR Green І Fluorescent Quantitative PCR for IBV and NDV.** Amplification curve of the IBV qPCR method.Melting curve of the IBV qPCR method.Standard curve of the IBV qPCR method. Amplification curve of the NDV qPCR method. Melting curve of the NDV qPCR method. Standard curve of the NDV qPCR method.

**Additional file 4. Detection of adenovirus shedding in the environment and identification of S1-HN protein expression in HD11 cells.** IFA identification of target protein PAD-S1-HN expression in HD11 cell lines. Western blot analysis of target protein PAD-S1-HN expression in HD11 cell lines, the empty adenovirus PAD control, PAD-S1-HN, the recombinant adenovirus PAD-S control, and the normal cell control. PCR detection of adenovirus shedding in the environment after PAD-S1-HN immunization. Lane 1 is a positive control; Lanes 3-6 represent mixed water and feed samples from the 1^st^ to the 4^t^^h^ week after the first immunization; Lanes 7-10 represent mixed fecal samples from the 1^st^ to the 4^th^ week after the first immunization, with each lane containing three pooled samples.

## Data Availability

All the data generated or analysed during this study are included in this published article and its supplementary information files.
