# Supplementary material for: A novel chimpanzee adenovirus vector vaccine for protection against infectious bronchitis and Newcastle disease in chickens
Source: Vet Res. 2025 May 16;56:100. doi: 10.1186/s13567-025-01528-6 (PMC12083102; doi:10.1186/s13567-025-01528-6)
Supplement: Supplementary file 1 — Additional file 1. Optimized Sequences of IBV S1 and NDV HN Proteins. [file 13567_2025_1528_MOESM1_ESM.doc]

**Optimization Report**

**Sequence optimization information:**

Expression System:*Homo sapiens,Gallus gallus*

Gene Length:3393 (bp)

**1.Codon Used Adjustment**

The best value is 1 for sequence optimization.


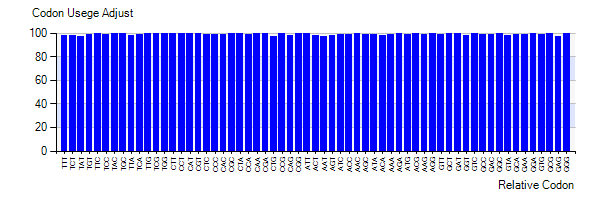


CAI: 0.68

Before Codon Adjustment


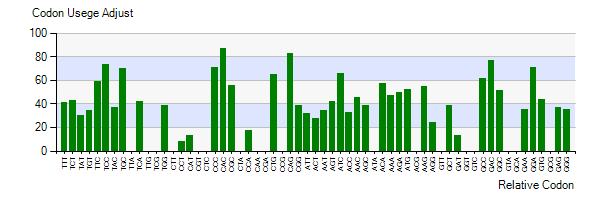


CAI: 0.96

After Codon Adjustment

**2.Codon Used Distribution**

Show the relative codon used distribution


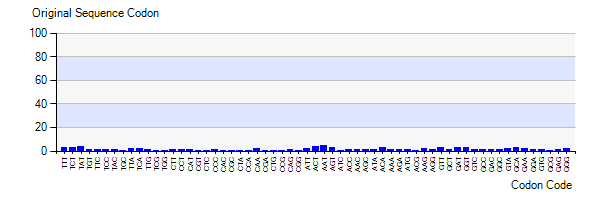


Before Optimization


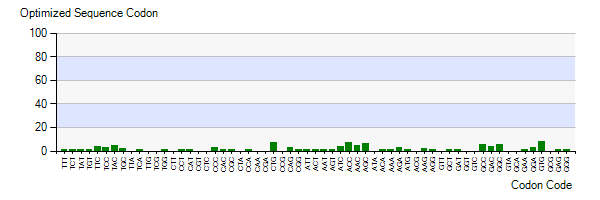


After Optimization

**3.GC Content:**

The comparison of GC content between original sequence and optimized sequence


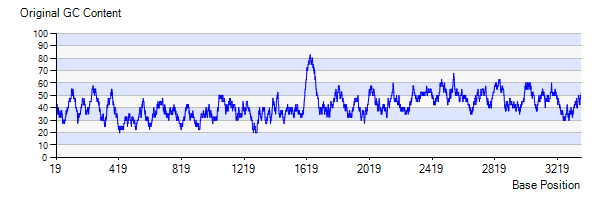


Before Optimization


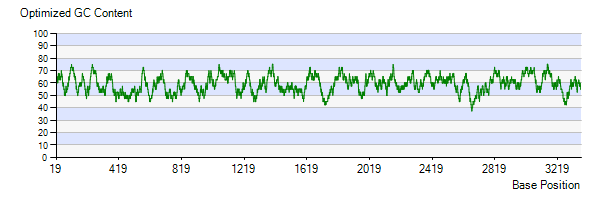


After Optimization

**4.Restriction Enzyme**

| Restriction Name | Original | Optimization |
| --- | --- | --- |
| KpnI | 0 | 0 |
| XbaI | 0 | 0 |

**5.Removed Repeats**

Before Optimization

Max Direct Length:12

After Optimization

Max Direct Length:14

**6.Optimized Sequence**

ATGCTGGGCAAGAGCCTGTTCCTGGTGACCATCCTGTGCGCCCTGTGCTCCGCCAACCTGTTCGACAGCGCCAACAACTACGTGTACTACTACCAGAGCGCCTTCAGGCCCCCCAACGGCTGGCACCTGCAGGGAGGAGCCTATGCCGTGGTGAACTCCACCAATTACACAAGCAACGCCGGCTCCGCCAGCGAATGCACCGTGGGAATCATCAAAGACGTGTACAACCAGAGCGCCGCTAGCATCGCCATGACCGCCCCCCCTCAGGGCATGGCTTGGAGCAGAAGCCAGTTCTGTTCAGCCCACTGCAACTTCAGCGAGATCACCGTGTTCGTGACTCACTGCTACTCATCCGGCGCCGGCTCTTGCCCCATCACCGGAATGATTGCCAGAGACCACATCAGAATCTCCGCCATGAAAAACGGGAGCCTGTTCTACAACCTGACCGTGTCTGTGAGCAAGTACAGCAGGTTTAAGAGCTTCCAGTGCGTGAACAATCTGACCTCCGTGTATCTGAACGGCGACCTGGTGTTCACATCCAATAAAACCACCGACGTGACCTCCGCTGGCGTGTACTTCAAGGCCGGCGGCCCCGTGAACTATAGCGTGATGAAGGAATTCAAGGTGCTGGCCTACTTTGTGAACGGGACTGCCCAGGACGTGATCCTGTGTGACAACAGCCCCAAGGGCCTGCTGGCCTGCCAGTACTCCACCGGAAACTTCTCTGACGGATTTTACCCCTTCACCAACTCCACCCTGGTGAGAGACAAATTCATTGTGTACCGCGAGAGCTCCGTGAACACAACCCTGACCCTGACCAACTTCACCTTCACCAACGTGTCCACTGCCCAGCCCAACAGCGGCGGCGTGTCAACCTTCCACCTGTACCAGACCCAGACCGCCCAGTCCGGCTATTACAACTTCAACCTGAGCTTCCTGAGCCAGTTCGTGTACAAGGCCTCCGATTTCATGTACGGCAGTTACCATCCCCGCTGCAGCTTCCGCCCCGAGACCATCAATTCCGGCCTGTGGTTCAACTCCCTGTCCGTGTCCCTGACCTACGGCCCCCTGCAGGGCGGATGCAAGCAGAGCGTGTTCTCCGGCAAGGCCACCTGCTGCTACGCCTACAGCTACAACGGCCCCCGGGCCTGCAAAGGCGTGTACTCTGGCGAACTGAGCAAAACCTTCGAATGCGGACTGCTGGTGTACGTGACTAAGTCTGACGGAAGCCGGATCCAGACCCGGACCGAACCCCTGGTGCTGACTCAGCACAATTACAACAACGTGACCCTGGACAAATGCGTGGACTACAACATCTACGGCAGGGTGGGCCAGGGCTTCATTACCAACGTGACCGACTCTGCCGCCAACTTCTCCTACCTGGCCGACGGCGGCCTGGCCATCCTGGATACCAGCGGGGCTATTGACGTGTTCGTGGTGCAGGGGAGCTACGGCTTCAACTACTACAAGGTGAACCCCTGTGAGGACGTGAACCAGCAGTTTGTGGTGTCTGGCGGCAACATTGTGGGCATCCTGACCAGCAGAAATGAGACAGGAAGCGAGCAGGTGGAGAACCAGTTCTACGTGAAGCTGACCAACAGCAGCCATAGAAGAAGAAGAGGGGGGGGAGGAAGCGGCGGAGGAGGATCAGGAGGAGGAGGAAGCGACAGAGCCGTGAGCCAGGTGGCCCTGGAGAACGACGAGAGAGAGGCCAAGAACACATGGAGACTGATCTTCAGAATTGCCATCCTGTTTCTGACAGTGGTGACACTGGCTATCTCAGTGGCCAGTCTGCTGTACAGTATGGGCGCCAGCACCCCAAGCGACCTGGTGGGAATCCCCACCCGGAACAGCCGGGCCGAGGAGAAAATCACCAGCACCCTGGGAAGCAACCAGGACGTGGTGGACCGCATCTACAAGCAGGTGGCCCTGGAAAGCCCACTGGCCCTGCTGAAAACCGAAACCACAATCATGAACGCCATCACCAGCCTGAGCTACCAGATCAACGGCGCCGCCAATAACAGCGGCTGGGGCGCTCTGATCCACGACCCCGACTACATCGGAGGCATCGGCAAAGAACTGATCGTGGACGACGCCAGCGACGTGACCTCATTCTACCCCAGCGCTTTCCAGGAGCACCTGAACTTTATCCCCGCCCCTACCACCGGCAGCGGCTGTACACGCATCCCCTCCTTTGACATGTCTGCCACCCACTACTGCTACACCCACAACGTGATCCTGTCTGGCTGCAGAGACCACTCCCACTCCTACCAGTACCTGGCCCTGGGAGTGCTGAGAACCTCCGCCACCGGCAGAGTGTTTTTTTCCACCCTGCGCAGCATCAACCTGGACGACACCCAGAACAGGAAGAGCTGCTCCGTGTCCGCCACCCCCCTGGGATGCGACATGCTGTGCAGCAAGGTGACCGAAACCGAGGAGGAGGACTACAACAGCGCCGTGCCCACCAGAATGGTGCACGGCAGACTGGGCTTTGACGGCCAGTACCACGAGAAGGACCTGGACGTGACCACCCTGTTCGGGGACTGGGTGGCTAACTATCCCGGCGTGGGAGGAGGAAGTTTCATCGACAGTAGAGTGTGGTTCTCAGTGTACGGAGGCCTGAAGCCCAACAGCCCCAGCGATACCGTGCAGGAGGGAAAGTACGTGATCTACAAGAGATACAATGATACATGCCCCGACGAACAGGACTACCAGATCAGAATGGCCAGAAGCAGTTACAAGCCCGGCAGATTCGGAGGGAAAAGAATCCAGCAGGCCATCCTGAGCATCAAGGTGAGCACCAGCCTGGGCGAAGACCCAGTGCTGACCGTGCCCCCCAACACCGTGACCCTGATGGGAGCCGAAGGAAGAATCCTGACCGTGGGCACCAGCCACTTCCTGTACCAGAGAGGCAGCAGCTACTTCAGTCCCGCCCTGCTGTACCCCATGACCGTGAGCAACAAGACAGCCACACTGCACAGCCCCTACACCTTCAACGCCTTCACCCGCCCCGGCTCCATCCCCTGTCAGGCTAGCGCCAGATGCCCCAACCCCTGCGTGACCGGCGTGTACACCGACCCACACCCCCTGATCTTCTACAGAAACCACACCCTGAGAGGCGTGTTCGGCACCATGCTGGACGGCGTGCAGGCCAGACTGAACCCCGCCTCCGCCGTGTTCGACTCCACCTCCAGAAGCAGAATTACCCGGGTGTCCAGCAGCAGCACCAAAGCCGCTTACACCACCTCCACCTGCTTCAAAGTGGTGAAAACCAACAAAACCTACTGCCTGAGCATCGCCGAGATCAGCAACACCCTGTTCGGCGAATTCCGCATCGTGCCCCTGCTGGTGGAAATCCTGAAAGACGACGGCGTGAGAGAGGCCAGAAGCGGC

**7.Protein Alignment**

Original Protein

MLGKSLFLVTILCALCSANLFDSANNYVYYYQSAFRPPNGWHLQGGAYAVVNSTNYTSNAGSASECTVGIIKDVYNQSAASIAMTAPPQGMAWSRSQFCSAHCNFSEITVFVTHCYSSGAGSCPITGMIARDHIRISAMKNGSLFYNLTVSVSKYSRFKSFQCVNNLTSVYLNGDLVFTSNKTTDVTSAGVYFKAGGPVNYSVMKEFKVLAYFVNGTAQDVILCDNSPKGLLACQYSTGNFSDGFYPFTNSTLVRDKFIVYRESSVNTTLTLTNFTFTNVSTAQPNSGGVSTFHLYQTQTAQSGYYNFNLSFLSQFVYKASDFMYGSYHPRCSFRPETINSGLWFNSLSVSLTYGPLQGGCKQSVFSGKATCCYAYSYNGPRACKGVYSGELSKTFECGLLVYVTKSDGSRIQTRTEPLVLTQHNYNNVTLDKCVDYNIYGRVGQGFITNVTDSAANFSYLADGGLAILDTSGAIDVFVVQGSYGFNYYKVNPCEDVNQQFVVSGGNIVGILTSRNETGSEQVENQFYVKLTNSSHRRRRGGGGSGGGGSGGGGSDRAVSQVALENDEREAKNTWRLIFRIAILFLTVVTLAISVASLLYSMGASTPSDLVGIPTRNSRAEEKITSTLGSNQDVVDRIYKQVALESPLALLKTETTIMNAITSLSYQINGAANNSGWGALIHDPDYIGGIGKELIVDDASDVTSFYPSAFQEHLNFIPAPTTGSGCTRIPSFDMSATHYCYTHNVILSGCRDHSHSYQYLALGVLRTSATGRVFFSTLRSINLDDTQNRKSCSVSATPLGCDMLCSKVTETEEEDYNSAVPTRMVHGRLGFDGQYHEKDLDVTTLFGDWVANYPGVGGGSFIDSRVWFSVYGGLKPNSPSDTVQEGKYVIYKRYNDTCPDEQDYQIRMARSSYKPGRFGGKRIQQAILSIKVSTSLGEDPVLTVPPNTVTLMGAEGRILTVGTSHFLYQRGSSYFSPALLYPMTVSNKTATLHSPYTFNAFTRPGSIPCQASARCPNPCVTGVYTDPHPLIFYRNHTLRGVFGTMLDGVQARLNPASAVFDSTSRSRITRVSSSSTKAAYTTSTCFKVVKTNKTYCLSIAEISNTLFGEFRIVPLLVEILKDDGVREARSG

Optimized Protein

MLGKSLFLVTILCALCSANLFDSANNYVYYYQSAFRPPNGWHLQGGAYAVVNSTNYTSNAGSASECTVGIIKDVYNQSAASIAMTAPPQGMAWSRSQFCSAHCNFSEITVFVTHCYSSGAGSCPITGMIARDHIRISAMKNGSLFYNLTVSVSKYSRFKSFQCVNNLTSVYLNGDLVFTSNKTTDVTSAGVYFKAGGPVNYSVMKEFKVLAYFVNGTAQDVILCDNSPKGLLACQYSTGNFSDGFYPFTNSTLVRDKFIVYRESSVNTTLTLTNFTFTNVSTAQPNSGGVSTFHLYQTQTAQSGYYNFNLSFLSQFVYKASDFMYGSYHPRCSFRPETINSGLWFNSLSVSLTYGPLQGGCKQSVFSGKATCCYAYSYNGPRACKGVYSGELSKTFECGLLVYVTKSDGSRIQTRTEPLVLTQHNYNNVTLDKCVDYNIYGRVGQGFITNVTDSAANFSYLADGGLAILDTSGAIDVFVVQGSYGFNYYKVNPCEDVNQQFVVSGGNIVGILTSRNETGSEQVENQFYVKLTNSSHRRRRGGGGSGGGGSGGGGSDRAVSQVALENDEREAKNTWRLIFRIAILFLTVVTLAISVASLLYSMGASTPSDLVGIPTRNSRAEEKITSTLGSNQDVVDRIYKQVALESPLALLKTETTIMNAITSLSYQINGAANNSGWGALIHDPDYIGGIGKELIVDDASDVTSFYPSAFQEHLNFIPAPTTGSGCTRIPSFDMSATHYCYTHNVILSGCRDHSHSYQYLALGVLRTSATGRVFFSTLRSINLDDTQNRKSCSVSATPLGCDMLCSKVTETEEEDYNSAVPTRMVHGRLGFDGQYHEKDLDVTTLFGDWVANYPGVGGGSFIDSRVWFSVYGGLKPNSPSDTVQEGKYVIYKRYNDTCPDEQDYQIRMARSSYKPGRFGGKRIQQAILSIKVSTSLGEDPVLTVPPNTVTLMGAEGRILTVGTSHFLYQRGSSYFSPALLYPMTVSNKTATLHSPYTFNAFTRPGSIPCQASARCPNPCVTGVYTDPHPLIFYRNHTLRGVFGTMLDGVQARLNPASAVFDSTSRSRITRVSSSSTKAAYTTSTCFKVVKTNKTYCLSIAEISNTLFGEFRIVPLLVEILKDDGVREARSG

**8.DNA Alignment**

Original DNA

ATGTTGGGGAAGTCACTGTTTTTAGTGACCATTTTGTGTGCACTATGTAGTGCAAATTTGTTTGATTCTGCCAATAATTATGTGTACTACTACCAAAGTGCCTTTAGGCCTCCAAATGGATGGCATTTGCAAGGGGGTGCTTATGCAGTAGTGAATTCTACTAATTATACTAGTAATGCCGGTTCTGCAAGTGAGTGCACTGTTGGTATTATTAAGGACGTCTATAATCAAAGTGCGGCTTCCATAGCTATGACAGCACCTCCTCAGGGTATGGCTTGGTCTAGGTCACAATTTTGTAGTGCACACTGTAACTTTTCTGAAATTACAGTTTTTGTCACACATTGTTATAGTAGTGGTGCAGGGTCTTGCCCTATAACAGGCATGATTGCACGTGATCATATTCGTATTTCTGCAATGAAAAATGGTTCTTTATTTTATAACTTAACAGTTAGCGTATCTAAATACTCTAGGTTTAAGTCTTTTCAATGTGTTAACAACCTCACATCTGTTTATTTAAATGGTGATCTTGTTTTTACTTCCAATAAAACTACTGATGTTACGTCAGCAGGTGTGTATTTTAAAGCAGGTGGACCTGTAAATTATAGTGTTATGAAAGAATTTAAGGTTCTTGCTTACTTTGTTAATGGTACAGCACAAGACGTAATTTTGTGTGACAATTCCCCTAAGGGTTTGCTAGCCTGTCAATATAGTACTGGCAATTTTTCAGATGGCTTCTATCCTTTTACTAATAGCACTTTGGTTAGGGACAAGTTCATTGTCTATCGTGAAAGTAGTGTTAATACTACTTTGACGTTAACTAATTTCACTTTTACTAATGTAAGTACTGCACAGCCTAATAGTGGTGGTGTTAGTACTTTTCATCTATATCAAACACAAACAGCTCAGAGTGGTTATTATAATTTTAATTTGTCATTTCTGAGTCAGTTTGTGTATAAGGCAAGTGATTTTATGTATGGGTCTTATCATCCTAGGTGTTCTTTTAGACCAGAAACCATTAATAGTGGTTTATGGTTTAATTCCTTGTCAGTTTCTCTTACTTATGGACCCCTACAGGGAGGGTGTAAGCAATCTGTTTTTAGTGGTAAGGCAACGTGTTGTTATGCCTACTCTTATAATGGCCCTAGGGCATGTAAAGGTGTTTATTCAGGTGAATTAAGCAAGACTTTTGAATGTGGATTGCTGGTTTATGTTACTAAGAGTGATGGCTCTCGTATACAAACTAGAACAGAGCCCTTAGTATTAACGCAACACAATTATAATAATGTTACTTTAGATAAGTGTGTTGACTATAATATATATGGCAGAGTAGGCCAAGGTTTTATTACTAATGTGACTGATTCTGCTGCTAATTTTAGTTATTTAGCAGATGGTGGGTTAGCTATTTTAGATACTTCGGGTGCCATAGATGTCTTTGTTGTACAGGGCAGCTATGGTTTTAATTATTACAAGGTCAATCCTTGTGAAGATGTTAACCAACAGTTTGTAGTGTCTGGTGGTAATATAGTTGGCATTCTTACTTCCAGAAATGAAACAGGTTCTGAACAGGTTGAGAACCAGTTTTATGTTAAGTTAACCAATAGCTCACATCGTCGTAGGCGTGGTGGCGGTGGCTCGGGCGGAGGTGGGTCGGGTGGCGGCGGATCAGACCGCGCCGTTAGCCAAGTTGCGTTAGAGAATGATGAAAGAGAGGCAAAAAATACATGGCGCTTGATATTCCGGATTGCAATCTTATTCTTAACAGTAGTGACCTTGGCTATATCTGTAGCCTCCCTTTTATATAGCATGGGGGCTAGCACACCTAGCGATCTTGTAGGCATACCGACTAGGAATTCCAGGGCAGAAGAAAAGATTACATCTACACTTGGTTCCAATCAAGATGTAGTAGATAGGATATATAAGCAAGTGGCCCTTGAGTCTCCGTTGGCATTGTTAAAAACTGAGACCACAATTATGAACGCAATAACATCTCTCTCTTATCAGATTAATGGAGCTGCAAACAACAGTGGGTGGGGGGCACTTATCCATGACCCAGATTATATAGGGGGGATAGGCAAAGAACTCATTGTAGATGATGCTAGTGATGTCACATCATTCTATCCCTCTGCATTTCAAGAACATCTGAATTTTATCCCGGCGCCTACTACAGGATCAGGTTGCACTCGAATACCCTCATTTGACATGAGTGCTACCCATTACTGCTACACCCATAATGTAATATTGTCTGGATGCAGAGATCACTCACATTCATATCAGTATTTAGCACTTGGTGTGCTCCGGACATCTGCAACAGGGAGGGTATTCTTTTCTACTCTGCGTTCCATCAACCTGGACGACACCCAAAATCGGAAGTCTTGCAGTGTGAGTGCAACTCCCCTGGGTTGTGATATGCTGTGCTCGAAAGTCACGGAGACAGAGGAAGAAGATTATAACTCAGCTGTCCCTACGCGGATGGTACATGGGAGGTTAGGGTTCGACGGCCAGTACCACGAAAAGGACCTAGATGTCACAACATTATTCGGGGACTGGGTGGCCAACTACCCAGGAGTAGGGGGTGGATCTTTTATTGACAGCCGCGTATGGTTCTCAGTCTACGGAGGGTTAAAACCCAATTCACCCAGTGACACTGTACAGGAAGGGAAATATGTGATATACAAGCGATACAATGACACATGCCCAGATGAGCAAGACTACCAGATTCGAATGGCCAGGTCTTCGTATAAGCCTGGACGGTTTGGTGGGAAACGCATACAGCAGGCTATCTTATCTATCAAGGTGTCAACATCCTTAGGCGAAGACCCGGTACTGACTGTACCGCCCAACACAGTCACACTCATGGGGGCCGAAGGCAGAATTCTCACAGTAGGGACATCTCATTTCTTGTATCAACGAGGGTCATCATACTTCTCTCCCGCGTTATTATATCCTATGACAGTCAGCAACAAAACAGCCACTCTTCATAGTCCTTATACATTCAATGCCTTCACTCGGCCAGGTAGTATCCCTTGCCAGGCTTCAGCAAGATGCCCCAACCCGTGTGTTACTGGAGTCTATACAGATCCACATCCCCTAATCTTCTATAGAAACCACACCTTGCGAGGGGTATTCGGGACAATGCTTGATGGTGTACAAGCAAGACTTAACCCTGCGTCTGCAGTATTCGATAGCACATCCCGCAGTCGCATTACTCGAGTGAGTTCAAGCAGTACCAAAGCAGCATACACAACATCAACTTGTTTTAAAGTGGTCAAGACTAATAAGACCTATTGTCTCAGCATTGCTGAAATATCTAATACTCTCTTCGGAGAATTCAGAATCGTCCCGTTACTAGTTGAGATCCTCAAAGATGACGGGGTTAGAGAAGCCAGGTCTGGC

Optimized DNA

ATGCTGGGCAAGAGCCTGTTCCTGGTGACCATCCTGTGCGCCCTGTGCTCCGCCAACCTGTTCGACAGCGCCAACAACTACGTGTACTACTACCAGAGCGCCTTCAGGCCCCCCAACGGCTGGCACCTGCAGGGAGGAGCCTATGCCGTGGTGAACTCCACCAATTACACAAGCAACGCCGGCTCCGCCAGCGAATGCACCGTGGGAATCATCAAAGACGTGTACAACCAGAGCGCCGCTAGCATCGCCATGACCGCCCCCCCTCAGGGCATGGCTTGGAGCAGAAGCCAGTTCTGTTCAGCCCACTGCAACTTCAGCGAGATCACCGTGTTCGTGACTCACTGCTACTCATCCGGCGCCGGCTCTTGCCCCATCACCGGAATGATTGCCAGAGACCACATCAGAATCTCCGCCATGAAAAACGGGAGCCTGTTCTACAACCTGACCGTGTCTGTGAGCAAGTACAGCAGGTTTAAGAGCTTCCAGTGCGTGAACAATCTGACCTCCGTGTATCTGAACGGCGACCTGGTGTTCACATCCAATAAAACCACCGACGTGACCTCCGCTGGCGTGTACTTCAAGGCCGGCGGCCCCGTGAACTATAGCGTGATGAAGGAATTCAAGGTGCTGGCCTACTTTGTGAACGGGACTGCCCAGGACGTGATCCTGTGTGACAACAGCCCCAAGGGCCTGCTGGCCTGCCAGTACTCCACCGGAAACTTCTCTGACGGATTTTACCCCTTCACCAACTCCACCCTGGTGAGAGACAAATTCATTGTGTACCGCGAGAGCTCCGTGAACACAACCCTGACCCTGACCAACTTCACCTTCACCAACGTGTCCACTGCCCAGCCCAACAGCGGCGGCGTGTCAACCTTCCACCTGTACCAGACCCAGACCGCCCAGTCCGGCTATTACAACTTCAACCTGAGCTTCCTGAGCCAGTTCGTGTACAAGGCCTCCGATTTCATGTACGGCAGTTACCATCCCCGCTGCAGCTTCCGCCCCGAGACCATCAATTCCGGCCTGTGGTTCAACTCCCTGTCCGTGTCCCTGACCTACGGCCCCCTGCAGGGCGGATGCAAGCAGAGCGTGTTCTCCGGCAAGGCCACCTGCTGCTACGCCTACAGCTACAACGGCCCCCGGGCCTGCAAAGGCGTGTACTCTGGCGAACTGAGCAAAACCTTCGAATGCGGACTGCTGGTGTACGTGACTAAGTCTGACGGAAGCCGGATCCAGACCCGGACCGAACCCCTGGTGCTGACTCAGCACAATTACAACAACGTGACCCTGGACAAATGCGTGGACTACAACATCTACGGCAGGGTGGGCCAGGGCTTCATTACCAACGTGACCGACTCTGCCGCCAACTTCTCCTACCTGGCCGACGGCGGCCTGGCCATCCTGGATACCAGCGGGGCTATTGACGTGTTCGTGGTGCAGGGGAGCTACGGCTTCAACTACTACAAGGTGAACCCCTGTGAGGACGTGAACCAGCAGTTTGTGGTGTCTGGCGGCAACATTGTGGGCATCCTGACCAGCAGAAATGAGACAGGAAGCGAGCAGGTGGAGAACCAGTTCTACGTGAAGCTGACCAACAGCAGCCATAGAAGAAGAAGAGGGGGGGGAGGAAGCGGCGGAGGAGGATCAGGAGGAGGAGGAAGCGACAGAGCCGTGAGCCAGGTGGCCCTGGAGAACGACGAGAGAGAGGCCAAGAACACATGGAGACTGATCTTCAGAATTGCCATCCTGTTTCTGACAGTGGTGACACTGGCTATCTCAGTGGCCAGTCTGCTGTACAGTATGGGCGCCAGCACCCCAAGCGACCTGGTGGGAATCCCCACCCGGAACAGCCGGGCCGAGGAGAAAATCACCAGCACCCTGGGAAGCAACCAGGACGTGGTGGACCGCATCTACAAGCAGGTGGCCCTGGAAAGCCCACTGGCCCTGCTGAAAACCGAAACCACAATCATGAACGCCATCACCAGCCTGAGCTACCAGATCAACGGCGCCGCCAATAACAGCGGCTGGGGCGCTCTGATCCACGACCCCGACTACATCGGAGGCATCGGCAAAGAACTGATCGTGGACGACGCCAGCGACGTGACCTCATTCTACCCCAGCGCTTTCCAGGAGCACCTGAACTTTATCCCCGCCCCTACCACCGGCAGCGGCTGTACACGCATCCCCTCCTTTGACATGTCTGCCACCCACTACTGCTACACCCACAACGTGATCCTGTCTGGCTGCAGAGACCACTCCCACTCCTACCAGTACCTGGCCCTGGGAGTGCTGAGAACCTCCGCCACCGGCAGAGTGTTTTTTTCCACCCTGCGCAGCATCAACCTGGACGACACCCAGAACAGGAAGAGCTGCTCCGTGTCCGCCACCCCCCTGGGATGCGACATGCTGTGCAGCAAGGTGACCGAAACCGAGGAGGAGGACTACAACAGCGCCGTGCCCACCAGAATGGTGCACGGCAGACTGGGCTTTGACGGCCAGTACCACGAGAAGGACCTGGACGTGACCACCCTGTTCGGGGACTGGGTGGCTAACTATCCCGGCGTGGGAGGAGGAAGTTTCATCGACAGTAGAGTGTGGTTCTCAGTGTACGGAGGCCTGAAGCCCAACAGCCCCAGCGATACCGTGCAGGAGGGAAAGTACGTGATCTACAAGAGATACAATGATACATGCCCCGACGAACAGGACTACCAGATCAGAATGGCCAGAAGCAGTTACAAGCCCGGCAGATTCGGAGGGAAAAGAATCCAGCAGGCCATCCTGAGCATCAAGGTGAGCACCAGCCTGGGCGAAGACCCAGTGCTGACCGTGCCCCCCAACACCGTGACCCTGATGGGAGCCGAAGGAAGAATCCTGACCGTGGGCACCAGCCACTTCCTGTACCAGAGAGGCAGCAGCTACTTCAGTCCCGCCCTGCTGTACCCCATGACCGTGAGCAACAAGACAGCCACACTGCACAGCCCCTACACCTTCAACGCCTTCACCCGCCCCGGCTCCATCCCCTGTCAGGCTAGCGCCAGATGCCCCAACCCCTGCGTGACCGGCGTGTACACCGACCCACACCCCCTGATCTTCTACAGAAACCACACCCTGAGAGGCGTGTTCGGCACCATGCTGGACGGCGTGCAGGCCAGACTGAACCCCGCCTCCGCCGTGTTCGACTCCACCTCCAGAAGCAGAATTACCCGGGTGTCCAGCAGCAGCACCAAAGCCGCTTACACCACCTCCACCTGCTTCAAAGTGGTGAAAACCAACAAAACCTACTGCCTGAGCATCGCCGAGATCAGCAACACCCTGTTCGGCGAATTCCGCATCGTGCCCCTGCTGGTGGAAATCCTGAAAGACGACGGCGTGAGAGAGGCCAGAAGCGGC

**9.Codon Used Table**

TTT [760066] TCT [657007] TAT [527910] TGT [454162]

TTC [879628] TCC [761575] TAC [670749] TGC [549103]

TTA [331010] TCA [527890] TAA [42331] TGA [66223]

TTG [559834] TCG [193498] TAG [33390] TGG [568211]

CTT [570223] CCT [754905] CAT [467596] CGT [199291]

CTC [842391] CCC [850717] CAC [652794] CGC [451821]

CTA [306962] CCA [730805] CAA [534929] CGA [265099]

CTG [1716500] CCG [302661] CAG [1480716] CGG [490938]

ATT [696126] ACT [569687] AAT [735740] AGT [523819]

ATC [906372] ACC [813098] AAC [837702] AGC [846250]

ATA [328370] ACA [658407] AAA [1067877] AGA [527971]

ATG [958977] ACG [267048] AAG [1388961] AGG [518408]

GTT [484200] GCT [806624] GAT [954112] GGT [468024]

GTC [625055] GCC [1189881] GAC [1088378] GGC [957196]

GTA [308989] GCA [695184] GAA [1261810] GGA [717638]

GTG [1220158] GCG [324263] GAG [1721098] GGG [713281]
