# Supplementary material for: A novel chimpanzee adenovirus vector vaccine for protection against infectious bronchitis and Newcastle disease in chickens
Source: Vet Res. 2025 May 16;56:100. doi: 10.1186/s13567-025-01528-6 (PMC12083102; doi:10.1186/s13567-025-01528-6)
Supplement: Supplementary file 2 — Additional file 2. Pathological Changes and Scoring of Organs Post IBV and NDV Challenge. [file 13567_2025_1528_MOESM2_ESM.docx]

**Additional file 2 Histopathology of main organs and tissues from IBV and NDV challenged chickens.**

| Organization code | Organization name | Result | score |
| --- | --- | --- | --- |
| PBS | Trachea | necrosis of the mucosal submucosa (++)  inflammatory cell infiltration (+) | 2 |
| PAD | Trachea | necrosis of the mucosal lamina propria (+)  inflammatory cell infiltration (+) | 2 |
| PAD-S1-HN | Trachea | (-) | 0 |
| QX-NDV | Trachea | (-) | 0 |
| PBS | Kidney | tubular degeneration and necrosis (+)  inflammatory cell infiltration (+) | 2 |
| PAD | Kidney | tubular degeneration and necrosis (+++) | 2 |
| PAD-S1-HN | Kidney | (-) | 0 |
| QX-NDV | Kidney | (-) | 0 |
| PBS | Lung | Hemorrhage (+++)  Congestion (+) | 2 |
| PAD | Lung | Hemorrhage (+++)  Congestion (++) | 2 |
| PAD-S1-HN | Lung | Hemorrhage (+) | 1 |
| QX-NDV | Lung | Hemorrhage (+) | 1 |
| PBS | Spleen | Spleen follicle atrophy (++++)  Reduction in the number of lymphocytes (+++) | 3 |
| PAD | Spleen | Spleen follicle atrophy (++++)  Reduction in the number of lymphocytes (+++) | 3 |
| PAD-S1-HN | Spleen | Spleen follicle atrophy (++) | 1 |
| QX-NDV | Spleen | Spleen follicle atrophy (++) | 1 |

Note: (-) represents no pathological changes in the tissue; (+) represents mild tissue damage; (++) represents moderate tissue damage; (+++) represents severe tissue damage; (++++) represents very severe tissue damage. 0 = no lesions; 1 = mild lesions; 2 = moderate lesions; and 3 = severe lesions.
