# Supplementary figures and images for: A novel chimpanzee adenovirus vector vaccine for protection against infectious bronchitis and Newcastle disease in chickens
Source: Vet Res. 2025 May 16;56:100. doi: 10.1186/s13567-025-01528-6 (PMC12083102; doi:10.1186/s13567-025-01528-6)

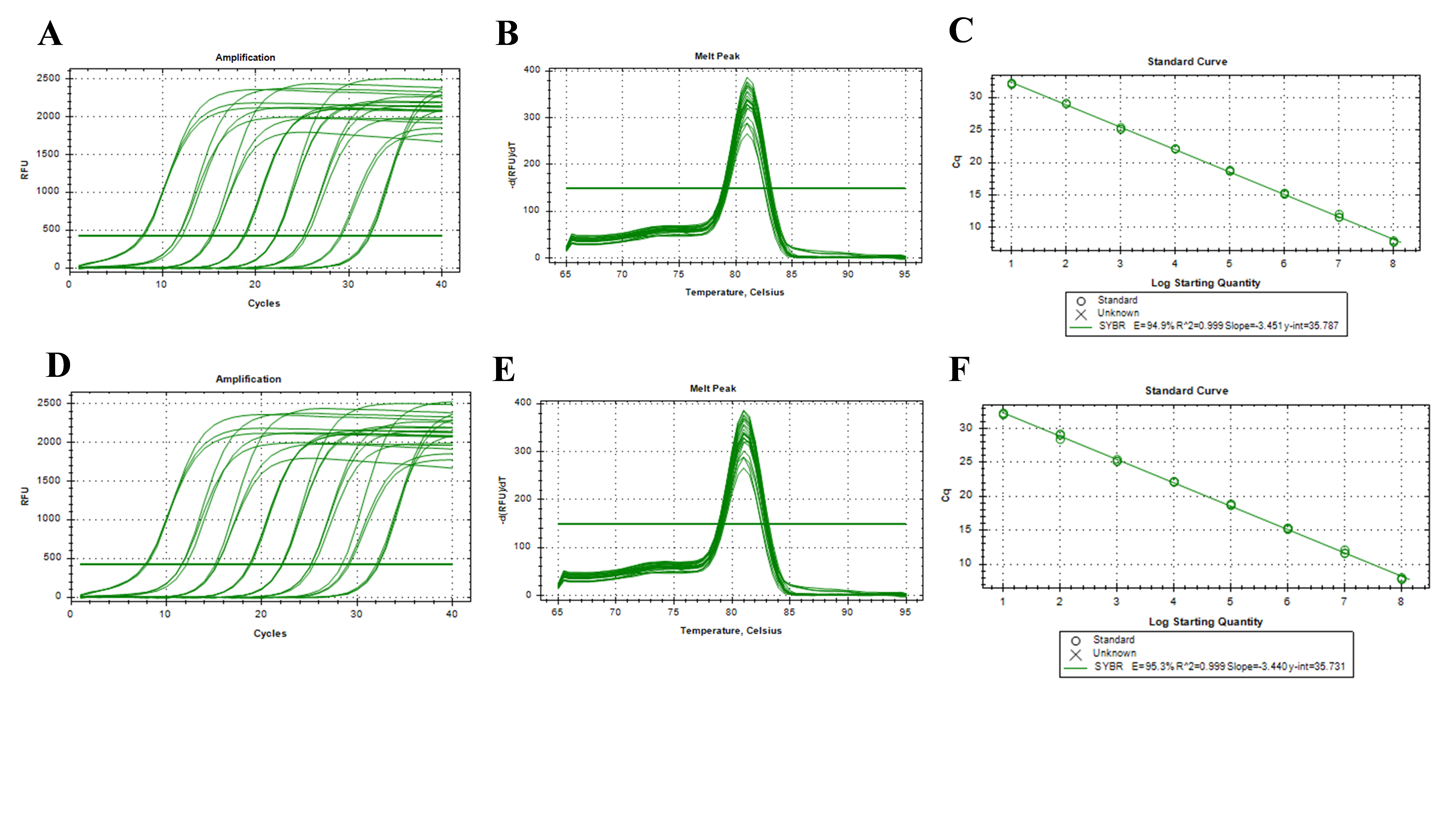

Supplement: Supplementary file 3 — Additional file 3. Establishment of SYBR Green І Fluorescent Quantitative PCR for IBV and NDV. Amplification curve of the IBV qPCR method.Melting curve of the IBV qPCR method.Standard curve of the IBV qPCR method. Amplification curve of the NDV qPCR method. Melting curve of the NDV qPCR method. Standard curve of the NDV qPCR method. [file 13567_2025_1528_MOESM3_ESM.tif]

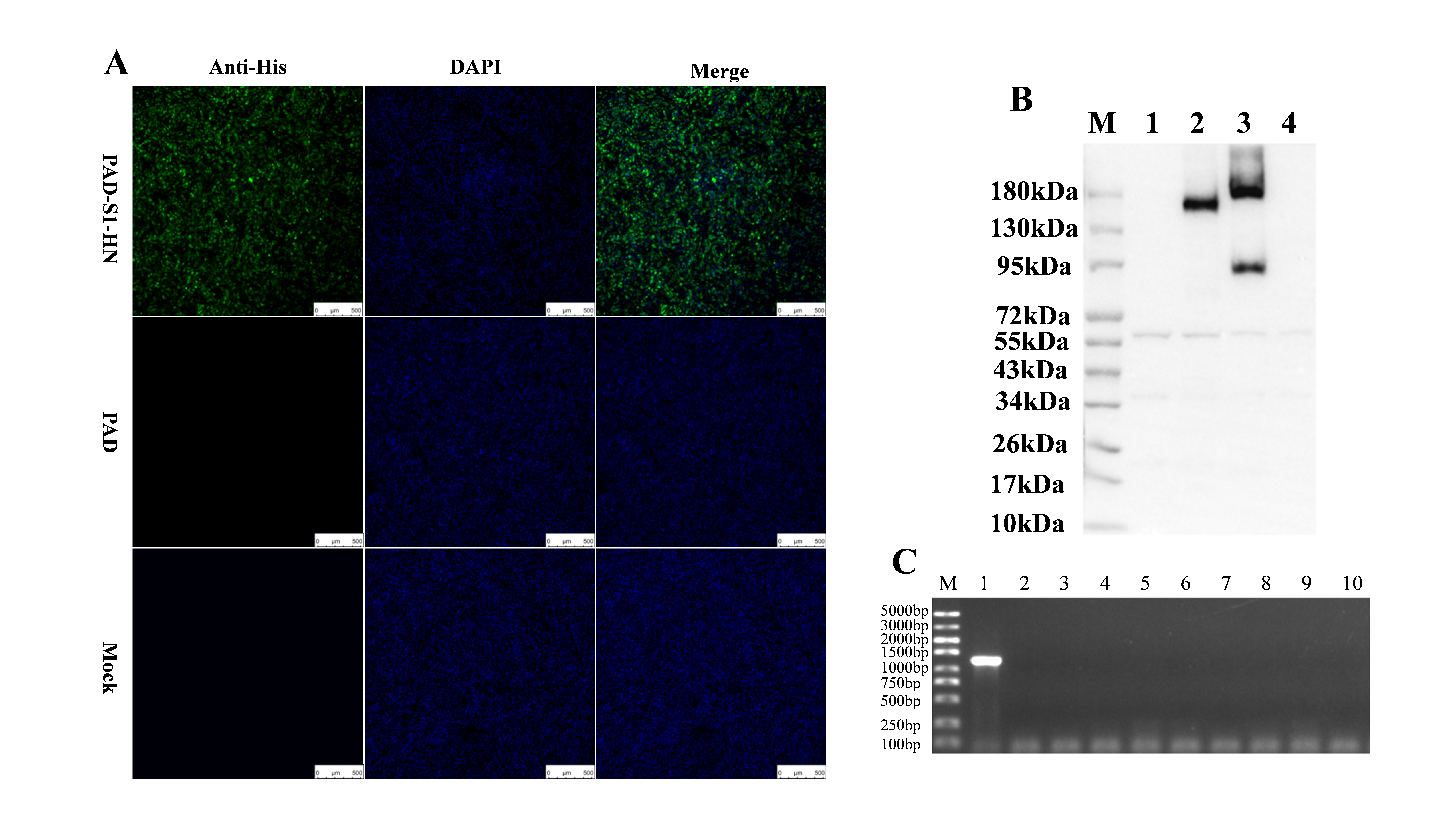

Supplement: Supplementary file 4 — Additional file 4. Detection of adenovirus shedding in the environment and identification of S1-HN protein expression in HD11 cells. IFA identification of target protein PAD-S1-HN expression in HD11 cell lines. Western blot analysis of target protein PAD-S1-HN expression in HD11 cell lines, the empty adenovirus PAD control, PAD-S1-HN, the recombinant adenovirus PAD-S control, and the normal cell control. PCR detection of adenovirus shedding in the environment after PAD-S1-HN immunization. Lane 1 is a positive control; Lanes 3-6 represent mixed water and feed samples from the 1st to the 4th week after the first immunization; Lanes 7-10 represent mixed fecal samples from the 1st to the 4th week after the first immunization, with each lane containing three pooled samples. [file 13567_2025_1528_MOESM4_ESM.tif]
